# Chemical and Biological Profiles of *Dendrobium* in Two Different Species, Their Hybrid, and Gamma-Irradiated Mutant Lines of the Hybrid Based on LC-QToF MS and Cytotoxicity Analysis

**DOI:** 10.3390/plants10071376

**Published:** 2021-07-05

**Authors:** Bomi Nam, Hyun-Jae Jang, Ah-Reum Han, Ye-Ram Kim, Chang-Hyun Jin, Chan-Hun Jung, Kyo-Bin Kang, Sang-Hoon Kim, Min-Jeong Hong, Jin-Baek Kim, Hyung-Won Ryu

**Affiliations:** 1 Advanced Radiation Technology Institute, Korea Atomic Energy Research Institute (KAERI), Jeongeup-si 56212, Jeollabuk-do, Korea; bomi1201@ncn.re.kr (B.N.); arhan@kaeri.re.kr (A.-R.H.); yrkim327@kaeri.re.kr (Y.-R.K.); chjin@kaeri.re.kr (C.-H.J.); shkim80@kaeri.re.kr (S.-H.K.); hongmj@kaeri.re.kr (M.-J.H.); jbkim74@kaeri.re.kr (J.-B.K.); 2 Institute of Natural Cosmetic Industry for Namwon, Namwon-si 55801, Jeollabuk-do, Korea; 3 Natural Medicine Research Center, Korea Research Institute of Bioscience & Biotechnology (KRIBB), Cheongju-si 28116, Chungbuk-do, Korea; water815@kribb.re.kr; 4 Jeonju AgroBio-Materials Institute, Jeonju-si 54810, Jeollabuk-do, Korea; chjung@jami.re.kr; 5 Research Institute of Pharmaceutical Sciences, College of Pharmacy, Sookmyung Women’s University, Seoul 04310, Korea; kbkang@sookmyung.ac.kr

**Keywords:** *Dendrobium*, Orchidaceae, *D. nobile*, *D. candidum*, *D. nobile* × *candidum*, gamma-irradiated mutant, metabolomics, cytotoxicity

## Abstract

The *Dendrobium* species (Orchidaceae) has been cultivated as an ornamental plant as well as used in traditional medicines. In this study, the chemical profiles of Dendrobii Herba, used as herbal medicine, *Dendrobium* in two different species, their hybrid, and the gamma-irradiated mutant lines of the hybrid, were systematically investigated via ultra-performance liquid chromatography coupled with quadrupole time-of-flight mass spectrometry (UPLC-QToF MS). Among the numerous peaks detected, 17 peaks were unambiguously identified. Gigantol (**1**), (1*R*,2*R*)-1,7-hydroxy-2,8-methoxy-2,3-dihydrophenanthrene-4(1*H*)-one (**2**), tristin (**3**), (−)-syringaresinol (**4**), lusianthridin (**5**), 2,7-dihydroxy-phenanthrene-1,4-dione (**6**), densiflorol B (**7**), denthyrsinin (**8**), moscatilin (**9**), lusianthridin dimer (**10**), batatasin III (**11**), ephemeranthol A (**12**), thunalbene (**13**), dehydroorchinol (**14**), dendrobine (**15**), shihunine (**16**), and 1,5,7-trimethoxy-2-phenanthrenol (**17**), were detected in Dendrobii Herba, while **1**, **2**, and **16** were detected in *D. candidum*, **1**, **11**, and **16** in *D. nobile*, and **1**, **2**, and **16** in the hybrid, *D. nobile* × *candidum*. The methanol extract taken of them was also examined for cytotoxicity against FaDu human hypopharynx squamous carcinoma cells, where Dendrobii Herba showed the greatest cytotoxicity. In the untargeted metabolite analysis of 436 mutant lines of the hybrid, using UPLC-QToF MS and cytotoxicity measurements combined with multivariate analysis, two tentative flavonoids (M1 and M2) were evaluated as key markers among the analyzed metabolites, contributing to the distinction between active and inactive mutant lines.

## 1. Introduction

*Dendrobium* is a genus of mostly epiphytic and lithophytic orchids in the family *Orchidaceae* [1]. It is a very large genus, containing more than 1800 species that are found in diverse habitats throughout much of South, East, and Southeast Asia, and in many of the islands of the Pacific [1]. The genera with medicinal importance include *D*. *nobile*, *D*. *chrysanthum, D*. *officinale*, *D*. *loddigessi*, *D*. *fimbriatum* var. *oculatum*, *D*. *moniliforme*, and *D*. *candidum* [1,2]. These have been used as traditional folk remedies for the treatment of various diseases, such as chronic atrophic gastritis, diabetes, and cardiovascular disease [3]. Previous phytochemical studies on the *Dendrobium* species have resulted in the isolation of diverse types of compounds, such as alkaloids [4,5,6,7,8,9,10], bibenzyls [9,11,12,13,14,15,16,17,18,19], phenanthrenes [19,20,21,22,23,24,25,26,27,28,29,30,31], fluorenones [18,31], sesquiterpenoids [6,32,33], lignans [21,34], flavonoids [35,36], and polysaccharides [37,38,39]. These compounds have been reported as exhibiting neuroprotective [4,5], anticancer [6,10,11,12,13,14,15,16,17,25,26,27,34], antioxidant [18,21,32,36,37,38], anti-inflammatory [22,23,38], and immunomodulatory activities [39]. In particular, there have been many reports on the mechanisms of their anticancer actions, including apoptosis and cell migration [11,12,13,14,15,16,17].

**Figure 1:**
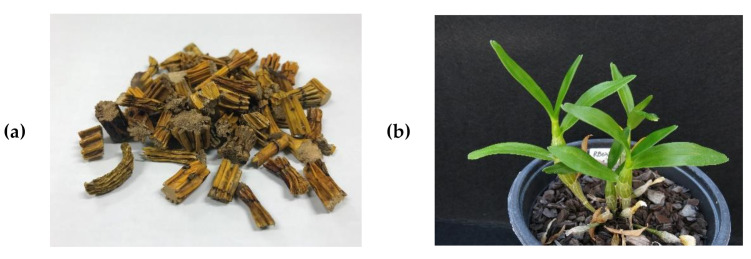

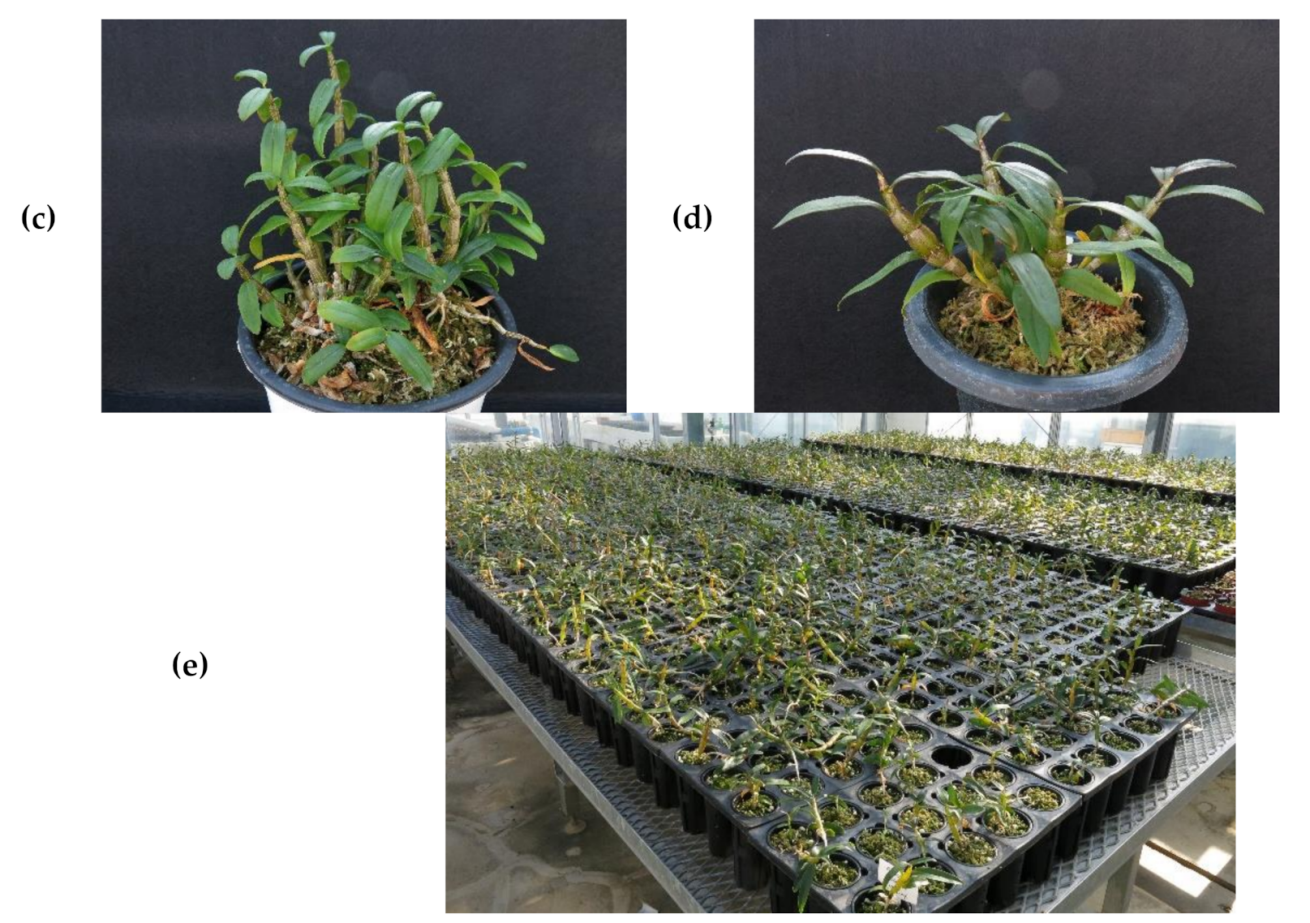
Pictures of the *Dendrobium* samples, (**a**) the medicinal herb Dendrobii Herba; (**b**) *D. nobile*; (**c**) *D. candidum*; (**d**) the hybrid *D. nobile* × *candidum*; (**e**) the gamma-irradiated mutant lines of *D. nobile* × *candidum*.

In our previous study, nine phenanthrenes, three bibenzyls, and a lignan were isolated from the ethyl acetate fraction of Dendrobii Herba, which were examined for their cytotoxicity against FaDu human hypopharynx squamous carcinoma cells [40]. Among them, densiflorol B, 6,7-dimethoxyphenanthrene-2,5-diol, dehydroorchinol, 1,5,7-trimethoxy-2-phenanthrenol, ephemeranthol A, and 3-[(1*E*)-2-(3-hydroxyphenyl)ethenyl]-5-methoxyphenol exhibited cytotoxicity, and together with moscatilin, exhibited remarkable activity (IC_50_, 2.55 μg/mL). In this study, electrospray ionization and the quadrupole time-of-flight mass spectrometry (ESI-QToF MS) data of these isolated compounds and five standards were obtained, as well as the composition of the methanol extracts of Dendrobii Herba, *D. nobile*, *D. candidum*, and the hybrid *D. nobile* × *candidum* (Figure 1). In addition, the cytotoxicity of these extracts against FaDu cells was evaluated.

Hybridization, genetic modification, or mutation using chemical or physical mutagens to improve the productivity and quality of plants have been widely used in plant breeding [41,42]. Mutation breeding following gamma irradiation has induced novel mutational traits while preserving the unique properties of the plant [43,44]. Our research group developed mutant lines of the hybrid *D. nobile* × *candidum* generated through the gamma irradiation (20 Gy) of its stems to produce mutants with enhanced biological activities and the improved yield of phytochemicals. Metabolomic studies of the *Dendrobium* species have previously been reported [45,46,47,48], however, there have been no studies on the chemical profile of *D. candidum*, the hybrid, *D. nobile* × *candidum*, and its gamma-irradiated mutant lines using LC-MS.

As part of our research project for the development of improved varieties, the chemical compositions of four *Dendrobium* extracts and their cytotoxicities against FaDu cells were compared, and mutant lines of the hybrid developed by radiation breeding were also evaluated for cytotoxicities with untargeted metabolite analysis. In addition, ultra-performance liquid chromatography coupled with quadrupole time-of-flight mass spectrometry (UPLC-QToF MS) and multivariate analysis, including principal component analysis (PCA) and orthogonal partial least squares analysis (OPLS-DA), were applied to characterize the metabolomic differences between the active and inactive mutant lines in FaDu cells.

## 2. Results and Discussion

### 2.1. Identification of the Compounds in Dendrobium Samples and Their Cytotoxic Activities

**Figure 2:**
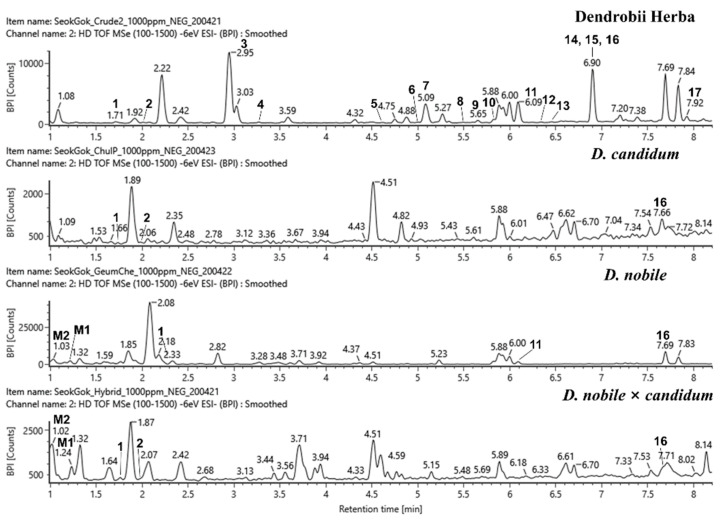
LC–MS base peak ion (BPI) chromatograms of four *Dendrobium* extracts at negative ion mode (6 eV, ESI^‒^). The selected chromatographic peaks are annotated with peak numbers referred to in Table 2.

The compounds **1**–**17** isolated from Dendrobii Herba [40] were ionized in both negative and positive ion modes using electrospray ionization (ESI). The QToF MS data are listed in Table 1. In addition, LC–MS base peak ion (BPI) chromatograms of all of the compounds and individual high-dimension mass spectra are provided in Figures S1–S17. The UPLC-QToF-MS analysis of the methanol extracts of the stems of four *Dendrobium* samples (Dendrobii Herba, *D. nobile*, *D. candidum*, and the hybrid *D. nobile* × *candidum*) was performed in the negative and positive ion modes (6 eV, ESI). Compared to their ESI^−^ and ESI^+^ MS chromatograms, the ESI^−^ of Dendrobii Herba, *D. candidum*, *D. nobile*, and *D. nobile* × *candidum* provided better sensitivity and lower detection limits compared to the ESI^+^ for the detection of the compounds, as shown in Figure 2. Among the numerous peaks detected, peaks 1‒9 and 11‒17 were unambiguously identified in Dendrobii Herba by comparing their retention times and masses with the previously acquired data of these compounds. Peak 10 gave a molecular ion at *m/z* 481.1639 [M − H]^−^, which is twice the molecular weight of lusianthridin (peak 5) and also exhibited the same fragment pathway as that of peak 5, suggesting that it was tentatively identified as a dimer of lusianthridin. Peak 10 was assumed to be phochinenin G or phochinenin D, as reported in *D. nobile*, because it had the same molecular weight and molecular formula as the lusianthridin dimer [19]. Most of the compounds were hardly detected in *D. nobile*, *D. candidum*, and *D. nobile* × *candidum* (Table 2). *D. nobile* contained three peaks: 1, 11, and 16. Specifically, peaks 1, 2, and 16 were detected in *D. candidum* and *D. nobile* × *candidum*. In previous studies, gigantol (peak 1), moscatilin (peak 9), and dendrobine (peak 15) have been identified in both *D. nobile* [6,18] and *D. candidum* [49,50], whereas densiflorol B (peak 7), denthyrsinin (peak 8), thunalbene (peak 13), (1*R*,2*R*)-1,7-hydroxy-2,8-methoxy-2,3-dihydrophenanthrene-4(1*H*)-one (peak 2), and 2,7-dihydroxy-phenanthrene-1,4-dione (peak 6), which were isolated as new compounds in our previous study [40], have never been reported in either plant. Tristin (peak 3), lusianthridin (peak 5), batatasin III (peak 11), ephemeranthol A (peak 12), dehydroorchinol (peak 14), shihunine (peak 16), and 1,5,7-trimethoxy-2-phenanthrenol (peak 17) have been found in *D. nobile* [19,22,51], but have not been reported in *D. candidum*. The metabolite identification of the hybrid, *D. nobile* × *candidum*, was reported for the first time in this study.

In addition, the cytotoxicity of the methanol extracts from four *Dendrobium* stems against the human pharynx squamous carcinoma (FaDu) cell line was also determined (Table 2 and Figure S20). The methanol extract of Dendrobii Herba showed the greatest activity, with an IC_50_ value of 63.03 μg/mL. The activities of the other *Dendrobium* samples exhibited IC_50_ values ranging from 84.47 to 91.93 μg/mL, with the hybrid *D. nobile* × *candidum* showing the lowest activity. In addition, the methanol extracts of the stems of 436 mutant lines of *D. nobile* × *candidum* were prepared and tested for cytotoxic ability against FaDu cells at a concentration of 50 μg/mL. The samples that showed less than 50% cell viability were determined as active samples (Table S1).

In our previous study of Dendrobii Herba [40], phytochemical investigation on the ethyl acetate-soluble fraction that showed cytotoxicity against FaDu cells resulted in the isolation of the active compounds, densiflorol B (peak 7), moscatilin (peak 9), ephemeranthol A (peak 12), dehydroorchinol (peak 14), and 1,5,7-trimethoxy-2-phenanthrenol (peak 17). These peaks were only detected in Dendrobii Herba and not in *D. nobile*, *D. candidum*, and their hybrid. Therefore, this result suggests that the distribution of the various constituents of Dendrobii Herba contributed more to its potent biological activity than the chemical composition of the other *Dendrobium* samples. In our further study on the mechanism of action of the most active compound, moscatilin, we have confirmed that moscatilin induced apoptosis in FaDu cells through the extrinsic and intrinsic apoptotic signaling pathways and the c-Jun N-terminal kinase (JNK) signaling pathway [17]. *D. nobile* constituents have been shown to exert anticancer activities: dendrobine has been shown to enhance the anticancer effect of cisplatin on non-small cell lung cancer cells via JNK stress signaling [10] and to induce apoptosis and inhibit cancer cell invasion in human gastric cells [52]. Nudol has been shown to inhibit the cell proliferation of U2OS osteosacorma cells by inducing cell cycle arrest at the G2/M phase and the migration of U2OS cells, and inducing cell apoptosis through the caspase-dependent pathway [53]. The extract of *D.* *candidum* has been reported to decrease the cell viability of human breast cancer cells by inducing cell cycle arrest at the G2/M phase and regulating the biomarkers (ERα, PGR, and GATA3) and oncogenes (p53, Ki67, and ELF5) [54]. To the best of our knowledge, the cytotoxicities of the methanol extracts from *D. nobile*, *D. candidum*, and *D. nobile* × *candidum* against FaDu cells were reported for the first time.

### 2.2. Untargeted UPLC-QToF MS Analysis of Gamma-Irradiated Mutant Lines of D. Nobile × Candidum

**Figure 3:**
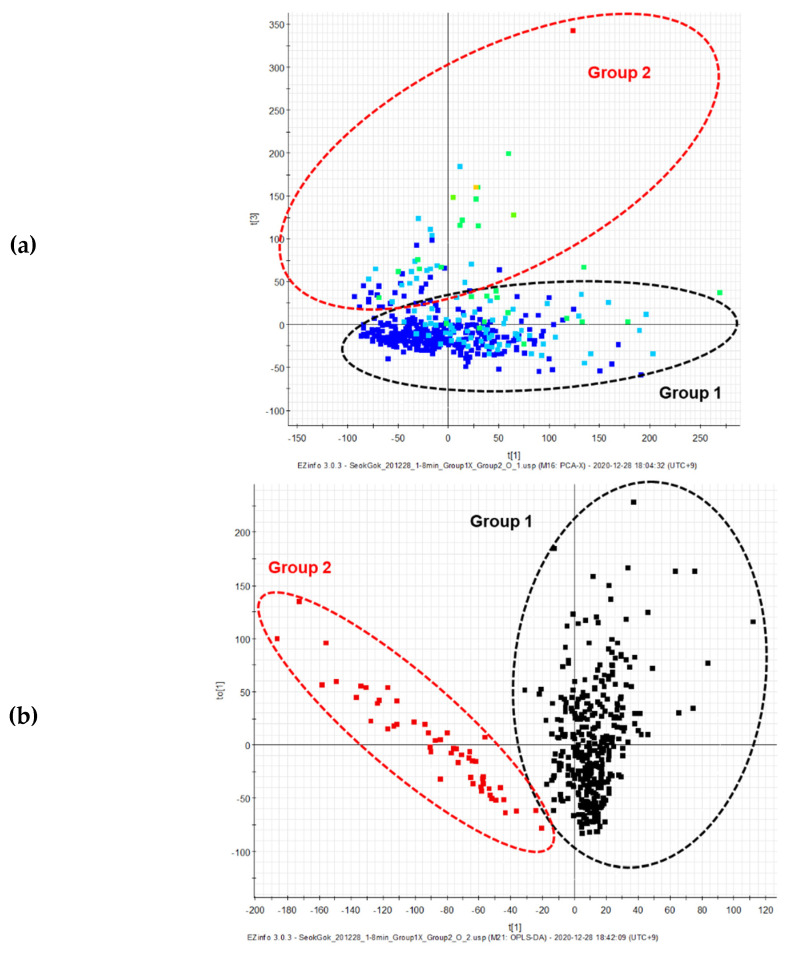

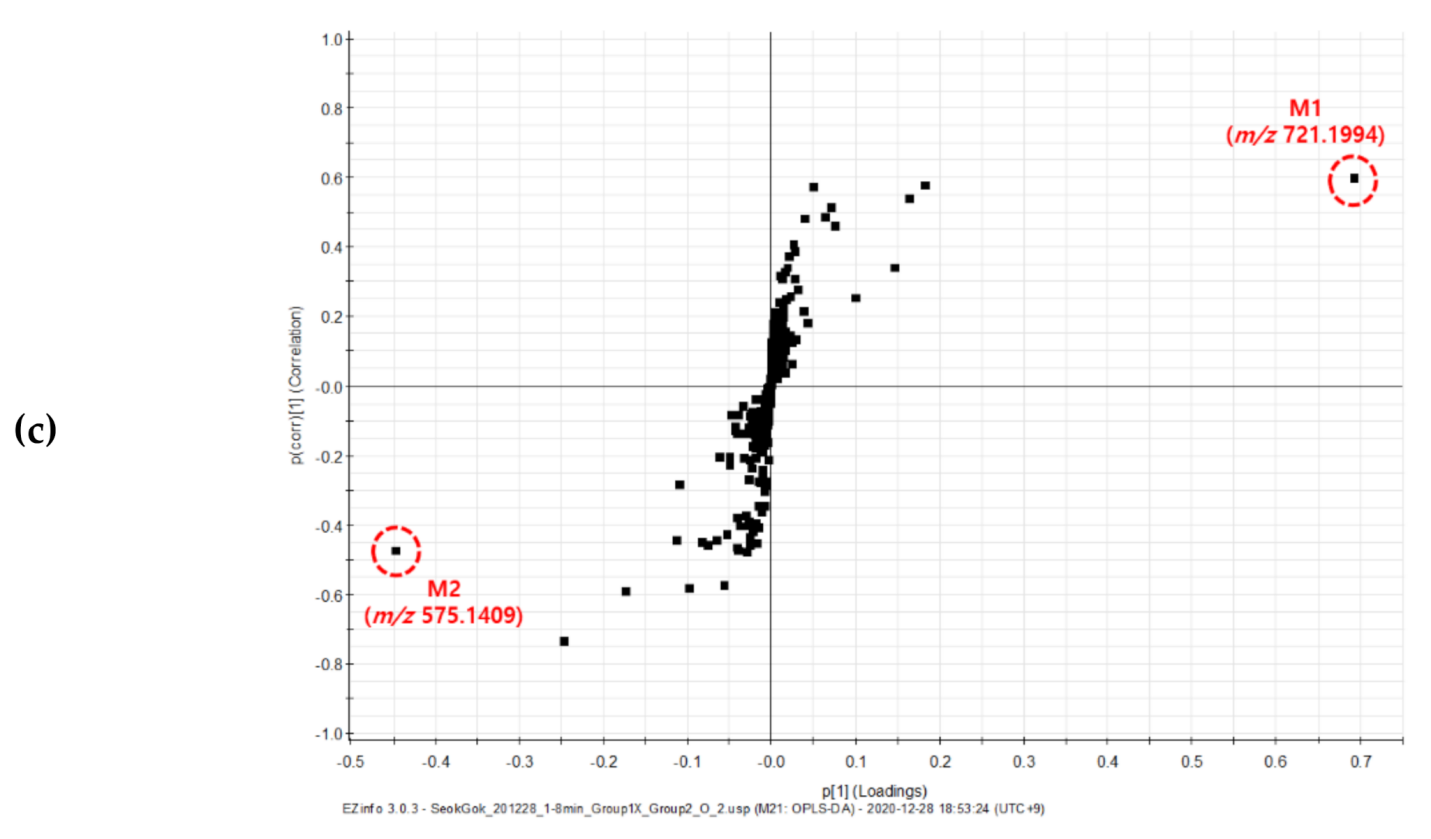
Principal component analysis (PCA) (**a**) and orthogonal partial least squares-discriminant analysis (OPLS-DA) (**b**) score plots and (**c**) *S*-plots of the inactive and active samples analyzed via UPLC-QToF MS.

Recently, multivariate statistical analysis has been shown to be an effective approach in distinguishing differences between experimental setups, in untargeted and targeted metabolomic studies [55]. First, the metabolite profiles of 436 mutant lines grown under the same environmental conditions after γ-irradiation with the same dose as the stems of *D. nobile* × *candidum* were analyzed via UPLC-QToF MS. Second, we performed principal component analysis (PCA), orthogonal partial least squares-discriminant analysis (OPLS-DA), and *S*-plots, which have been widely used in the field in recent years for the metabolomic analysis of extremely complex samples. There were no significant differences among the mutant lines in the chromatograms (data not shown), however, PCA and OPLS-DA score plots showed a clear separation of the clusters when applying multivariate analysis to the normalized dataset (Figure 3). In particular, supervised OPLS-DA has been widely used to study the differences between two similar groups. The OPLS-DA model quality can be estimated using the cross-validation parameters Q^2^ (model predictability) and R^2^(*y*) (total explained variation for the X matrix). OPLS-DA of the samples produced one predictive and one orthogonal (1 + 3) component and showed that the cross-validated predictive ability Q^2^ was 0.684, and the variance related to the differences between the two origins R^2^(*y*) was 0.693. In most cases, a Q^2^ value greater than 0.5 is adequate, and the difference between R^2^ and Q^2^ values should be less than 0.3. Third, the *S*-plot (Point, *t_R_*-*m/z* pair) from the OPLS-DA model, which is a useful tool for comparing the magnitude and reliability of a variable, was also analyzed. The markers associated with the mutant lines of *D. nobile* × *candidum* were based on the threshold of variable importance in the projection (VIP) value (VIP > 1.0) from the *S*-plot. The two identified metabolites, M1 and M2, had VIP values greater than 1.0, making them the primary markers of the differences between the two clusters for the mutant lines of *D. nobile* × *candidum* on the OPLS-DA score plot (Figure 3).

Marker ions ([M − H]^−^) at *m/z* 721.1994 (M1) were located far from the center and were in the same direction as group 1, indicating distinguishable markers for group 1. Differential markers in the same direction as group 2 demonstrated molecular ions ([M − H]^−^) at *m/z* 575.1409 (M2). These markers were identified as unknown metabolites that did not match with the compounds reported here (**1**‒**17**) and any compounds previously reported in the *Dendrobium* species. M1, which belongs to the inactive group, gave a molecular ion at *m/z* 721.1994 [M – H]^–^ corresponding to the molecular formula C_33_H_38_O_18_ and −0.56 ppm of *m/z* error (Figure S18). The marker M1 (*t_R_* = 1.03 min) was classified as the 3-hydroxy-3-methylglutaryl (HMG) moiety of *C*-glucoside and *C*-rhamnoside with MS/MS fragmentation patterns (*m/z* 577, 487, 457, 383, and 353) corresponding to the loss of HMG (*m/z* 144), *C*-glucoside (*m/z* 90 and 120), and *C*-rhamnoside (*m/z* 74 and 104) fragments. Thus, the M1 marker was tentatively identified as apigenin 6-*C*-α-L-rhamnopyranoside-8-*C*-[6′’-*O*-(3-hydroxy-3-methylglutaryl)]-β-D-glucopyranoside [56]. Another marker, M2, in the active group, demonstrated a molecular ion at *m/z* 575.1409 [M – H]^–^, corresponding to a molecular formula C_27_H_28_O_14_ and -0.38 ppm of *m/z* error (Figure S19). The marker M2 (*t_R_* = 1.21 min) was classified as the 3-hydroxy-3-methylglutaryl (HMG) moiety and *O*-glucoside with MS/MS fragmentation patterns (*m/z* 431 and 269) corresponding to the loss of the HMG (*m/z* 144) and *O*-glucoside (*m/z* 162) fragments. Thus, the M2 marker was tentatively identified as apigenin 7-*O*-[6′′-*O*-(3-hydroxy-3-methylglutaryl)]β-d-glucopyranoside [57].

Multivariate analysis clearly differentiated the samples into two clusters depending on their activities: a group of 384 extracts without cytotoxicity (Group I) and a group of 52 extracts (Group II) showing less than 50% cell viability (Figure 3). These results correlated with different metabolites and active components. *C*-glycosylation or *O*-glycosylation of apigenin may affect its metabolism, and in turn, affect both its anticancer potential and biological benefits. Previous studies on flavonoids have demonstrated that the sugars of *C*- and/or *O*-glycosides have potent anticancer properties [58,59]. These results are consistent with those published elsewhere, which also found that these metabolites were the major compounds present in the gamma-irradiated mutant lines of *D. nobile* × *candidum*.

## 3. Materials and Methods

### 3.1. General

UPLC-QToF MS was performed using a Waters ACQUITY UPLC I-Class system combined with a Vion IMS QToF mass spectrometer (Waters, Milford, MA, USA), equipped with an ACQUITY UPLC BEH C18 column (2.1 mm × 100 mm i.d., 1.7 μm; Waters). All data were processed using UNIFI software (v1.9, Waters). A (^60^Co)-irradiator (150 TBq capacity; AECL, Ottawa, ON, Canada) was used for gamma irradiation. Compounds **1**‒**12** were isolated from Dendrobii Herba (the stems of the *Dendrobium* species), as described in our previous study [40]. The standard compounds, dendrobine (**13**), batatasin III (**16**), and lusianthridin (**17**) were purchased from Wuhan ChemFaces Biochemical Co., Ltd. (Hubei, China). Shihunine (**14**) and tristin (**15**) were obtained from Wuhan ChemNorm Biotech Co., Ltd. (Hubei, China) and Chengdu Biopurify Phytochemicals Ltd. (Chengdu, China), respectively. All other chemicals and solvents used in this study were of analytical grade (J. T. Baker, Phillipsburg, NJ, USA).

### 3.2. Plant Materials

Dendrobii Herba stems (CK PHARM Co., Ltd., Seoul, Korea) were purchased from the Jewondang herb shop in Jeongup-si, Jeollabuk-do, Korea. *D. nobile*, *D. candidum*, the hybrid *D. nobile* × *candidum*, and its gamma-irradiated mutant lines were verified as authentic and grown by Dr. Sang Hoon Kim (Korea Atomic Energy Research Institute). The hybrids (*D. nobile* × *candidum*) were irradiated with a single dose of γ-rays (20 Gy) emitted from a labeled cobalt (^60^Co) source (150 TBq capacity; AECL) for 24 h at the Korea Atomic Energy Research Institute, Jeongeup-si, Korea. Approximately 1400 gamma-irradiated mutant lines derived from *D. nobile* × *candidum* were grown in a greenhouse under the same conditions as *D. nobile*, *D. candidum*, and *D. nobile* × *candidum* for two years (2017–2019). Two to three stems of these plant materials with good growth and yield (approximately 400 in total) were randomly collected in November 2019. The collected stems were dried using an air-drying method (air-conditioned at a temperature of 40 °C for 72 h). Voucher specimens of *D. nobile* (accession no. RB033), *D. candidum* (accession no. RB030), and the hybrid *D. nobile* × *candidum* (accession no. RB038) were deposited at the Advanced Radiation Technology Institute, Korea Atomic Energy Research Institute.

### 3.3. Sample Preparation

Dried stems of Dendrobii Herba, *D. nobile*, *D. candidum*, the hybrid *D. nobile* × *candidum*, and its gamma-irradiated mutant lines were chopped into small pieces. One gram of each stem was extracted with 30 mL of methanol using an ultrasonic bath for 60 min and was evaporated to produce the methanol extract. Each dried methanol extract (1 mg) was dissolved in 1 mL of methanol for UPLC-QTof MS analysis. Compounds **1**‒**17** were dissolved in methanol at a concentration of approximately 0.1 mg/mL. This sample solution was filtered through a 0.20-μm polyvinylidene fluoride filter for chromatographic analysis. For cytotoxicity evaluation, each dried methanol extract (1 mg) was initially dissolved in dimethyl sulfoxide at a concentration of 100 mg/mL. Subsequently, the final concentration was diluted to 1.5625, 3.125, 6.25, 12.5, 25, 50, and 100 μg/mL using a minimum essential medium (MEM; Corning, Manassas, VA, USA) for Dendrobii Herba, *D. nobile*, *D. candidum*, and the hybrid *D. nobile* × *candidum*, respectively, and 50 μg/mL for the gamma-irradiated mutant lines of the hybrid.

### 3.4. UPLC-QTof MS Analysis

The methanol extracts of Dendrobii Herba, *D. nobile*, *D. candidum*, the hybrid, *D. nobile* × *candidum*, and compounds (**1**‒**17**) were analyzed using a Waters ACQUITY UPLC I-Class system combined with a Vion IMS QToF mass spectrometer (Waters). Each sample (1000 ppm, 2 μL) was injected into an ACQUITY UPLC BEH C18 column (2.1 mm × 100 mm i.d., 1.7 μm; Waters). The temperature of the column oven was maintained at 35 °C. The flow rate was 0.4 mL/min using a mobile phase comprising 0.1% formic acid in water (*v/v*; solvent A) and 0.1% formic acid in acetonitrile (*v/v*; solvent B). Gradient elution was carried out as follows: 0–1.0 min, 23% B; 1.0–3.0 min, 23–27% B; 3.0–5.5 min, 27–37% B; 5.5–8.0 min, 37–62% B; 8.0–8.3 min, 62–100% B; 8.3–10.0 min, 100% B; 10.0–10.3 min, 100–23% B; 10.3–12.0 min, 23% B. The mass spectrometer was operated in negative or positive ion mode with the following parameters: source temperature, 110 °C; desolvation temperature, 350 °C; capillary voltage, 2300 V; cone voltage, 40 V; cone gas flow, 50 L/h; flow rate of desolvation gas (N_2_), 800 L/h; mass scan range, 100–1500 Da; scan time, 0.1 s. The full scan data, MS/MS spectra, accurate mass, and elemental composition were calculated using UNIFI software (Waters).

### 3.5. Cytotoxicity Assay

Human pharynx squamous carcinoma FaDu cells were purchased from the Korean Cell Line Bank (Seoul, Korea). These cells were cultured in a minimum essential medium (MEM; Corning, Manassas, VA, USA) supplemented with 10% heat-inactivated FBS (Hyclone, Logan, UT, USA) in a humidified incubator with 5% CO_2_ at 37 °C. To determine the viability of FaDu cells, a CCK-8 assay kit (Dojindo, Kumamoto, Japan) was used according to the manufacturer’s protocol. Briefly, FaDu cells were seeded into 96-well plates at a density of 2 × 10^4^ cells/mL and incubated at 37 °C for 24 h. After incubation, the cultured FaDu cells were treated with the indicated concentration of four *Dendrobium* extracts (1.5625−100 μg/mL) and the extracts of mutant lines (50 μg/mL) for 72 h. Thereafter, 10 μL of CCK-8 reagent was added to the cultured FaDu cells and then incubated for a further 4 h, after which the absorbance was measured at 450 nm using a SPARK^®^ multimode microplate reader (Tecan, Männedorf, Switzerland). Afterward, the 50% inhibitory concentration (IC_50_) values were calculated from a dose-response analysis performed using GraphPad Prism software (GraphPad Software, La Jolla, CA, USA).

### 3.6. Chemometric Data Analysis

The raw mass data were normalized to the total intensity (area) and analyzed using Progenesis^®^ QI software v2.4 (Waters). The parameters included a retention time range of 1.0−8.0 min, a mass range from 100 Da to 1,500 Da, and a mass tolerance of 0.01 Da. The isotopic data were excluded, the noise elimination level was 10, and the mass and retention time windows were 0.04 min and 0.1 min, respectively. After creating a suitable processing method, the dataset was processed using the Create dataset window. The resulting two-dimensional matrix for the measured mass values and intensities for each sample was further exported to EZinfo software v3.0.3 (Waters) using both unsupervised principal component analysis and supervised OPLS-DA.

## 4. Conclusions

In conclusion, the chemical compositions of Dendrobii Herba, *D. nobile*, *D. chrysanthum*, and their hybrid, *D. nobile* × *candidum*, were analyzed via UPLC-QToF MS and were identified by comparing the ESI QToF MS data of the isolates (**1**‒**13**) from Dendrobii Herba and the standards (**14**‒**17**). The results showed that the chemical compositions of each sample were different, demonstrating that each sample from different species could be well-distinguished, and the quality of the different samples could be determined through this method. The distribution and content of the constituents of each sample were estimated to be related to their cytotoxic activities against FaDu cells. In addition, UPLC-QToF MS combined with multivariate analysis was used to analyze the chemical profiles of the gamma-irradiated mutant lines derived from *D. nobile* × *candidum*. The results showed a clear separation of different groups of the mutant lines and the distinguished marker metabolites (M1 and M2), according to the two clusters. These markers were tentatively identified as apigenin 6-*C*-α-L-rhamnopyranoside-8-*C*-[6′′-*O*-(3-hydroxy-3-methylglutaryl)]-β-D-glucopyranoside and apigenin 7-*O*-[6′’-*O*-(3-hydroxy-3-methylglutaryl)]-β-D-glucopyranoside, respectively. Based on the cytotoxicity screening results, the samples were also divided into two groups (384 inactive samples and 52 active samples), where these markers corresponded to the two groups contributing to the correlation of the inactive and active extracts, respectively. This study was an attempt to select superior and differentiated mutant lines based on biochemical analyses. Therefore, these results will serve as a reference for future investigations of the mutation mechanism of the gamma-irradiated mutant lines and their quality evaluation for improved mutant selection.

## Figures and Tables

**Table 1:** ESI QToF MS data of compounds isolated from Dendrobii Herba and four standard compounds.

Peak No.	Identification	t*_R_* (min)	Observed *m/z* (Da)	Calculated *m/z* (Da)	Error (ppm)	Molecular Formula	Fragments
1	Gigantol	1.73	275.12688 [M + H]^+^	275.1278	−2.14	C_16_H_18_O_4_	198
2	(1*R*,2*R*)-1,7-Hydroxy-2,8-methoxy-2,3-dihydrophenanthrene-4(1*H*)-one	1.98	287.09214 [M − H]^−^	287.0914	4.44	C_16_H_14_O_5_	272, 239
3	Tristin	2.98	259.09822 [M − H]^‒^	259.0965	5.17	C_15_H_16_O_4_	243
4	(-)-Syringaresinol	3.32	417.15652 [M − H]^‒^	417.1555	5.21	C_22_H_26_O_8_	387, 190
5	Lusianthridin	4.69	241.08710 [M − H]^−^	241.087	−4.67	C_15_H_14_O_3_	116
6	2,7-Dihydroxy-phenanthrene-1,4-dione	5.02	239.03168 [M − H]^−^	239.0339	−9.26	C_14_H_8_O_4_	256
7	Densiflorol B	5.07	253.05075 [M − H]^−^	253.0495	4.11	C_15_H_10_O_4_	238
8	Denthyrsinin	5.49	299.09974 [M − H]^−^	299.0925	22.73	C_17_H_16_O_5_	284, 254
9	Moscatilin	5.63	305.13779 [M + H]^+^	305.1384	−3.52	C_17_H_20_O_5_	181
10	Lusianthridin dimer	5.84	481.16393 [M − H]^‒^	481.1657	−4.67	C_30_H_26_O_6_	116
11	Batatasin III	6.05	243.11115 [M − H]^‒^	243.1027	31.18	C_15_H_16_O_3_	-
12	Ephemeranthol A	6.31	273.11093 [M + H]^+^	273.1121	5.21	C_16_H_16_O_4_	272, 241, 213
13	Thunalbene	6.43	241.08710 [M − H]^‒^	241.087	1.33	C_15_H_14_O_3_	-
14	Dehydroorchinol	7.7	255.10002 [M + H]^+^	255.1016	−2.21	C_16_H_14_O_3_	240
15	Dendrobine	7.7	264.19496 [M + H]^+^	264.1958	−2.03	C_16_H_25_NO_2_	-
16	Shihunine	7.7	203.09751 [M]^+^	203.0952	11.49	C_12_H_13_NO_2_	405, 203
17	1,5,7-Trimethoxy-2-phenanthrenol	7.97	285.11172 [M + H]^+^	285.1121	−5.40	C_17_H_16_O_4_	253, 225

**Table 2:** Identification of individual compounds in extracts prepared from Dendrobii Herba, *D. nobile*, *D. candidum*, the hybrid, *D. nobile* × *candidum*, and their cytotoxicities against FaDu cells.

Samples	Cytotoxicity ^1^ (IC_50_, μg/mL)	Peak No.																
		1	2	3	4	5	6	7	8	9	10	11	12	13	14	15	16	17
Dendrobii Herba	63.03	+	+	+	+	+	+	+	+	+	+	+	+	+	+	+	+	+
*D. candidum*	84.47	+	+	−	−	−	−	−	−	−	−	−	−	−	−	−	+	−
*D. nobile*	89.22	+	−	−	−	−	−	−	−	−	−	+	−	−	−	−	+	−
*D. nobile* × *candidum*	91.93	+	+	−	−	−	−	−	−	−	−	−	−	−	−	−	+	−

^1^ Cisplatin as a positive control showed an IC_50_ value of 1.52 μM.

## Data Availability

Not applicable.

## Supplementary Materials

The following are available online at https://www.mdpi.com/article/10.3390/plants10071376/s1, Figure S1: ESI QTof MS spectrum of gigantol (peak 1), Figure S2: ESI QTof MS spectrum of (1*R*,2*R*)-1,7-hydroxy-2,8-methoxy-2,3-dihydrophenanthrene-4(1*H*)-one (peak 2), Figure S3: ESI QTof MS spectrum of tristin (peak 3), Figure S4: ESI QTof MS spectrum of (-)-syringaresinol (peak 4), Figure S5: ESI QTof MS spectrum of lusianthridin (peak 5), Figure S6: ESI QTof MS spectrum of 2,7-dihydroxy-phenanthrene-1,4-dione (peak 6), Figure S7: ESI QTof MS spectrum of densiflorol B (peak 7), Figure S8: ESI QTof MS spectrum of denthyrsinin (peak 8), Figure S9: ESI QTof MS spectrum of moscatilin (peak 9), Figure S10: ESI QTof MS spectrum of lusianthridin dimer (peak 10), Figure S11: ESI QTof MS spectrum of batatasin III (peak 11), Figure S12: ESI QTof MS spectrum of ephemeranthol A (peak 12), Figure S13: ESI QTof MS spectrum of thunalbene (peak 13), Figure S14: ESI QTof MS spectrum of dehydroorchinol (peak 14), Figure S15: ESI QTof MS spectrum of dendrobine (peak 15), Figure S16: ESI QTof MS spectrum of shihunine (peak 16), Figure S17: ESI QTof MS spectrum of 1,5,7-trimethoxy-2-phenanthrenol (peak 17), Figure S18: ESI QTof MS spectrum of differential metabolite (M1), Figure S19: ESI QTof MS spectrum of differential metabolite (M2), Figure S20: Cytotoxicities of the methanol extracts of Dendrobii Herba, *D. nobile*, *D. candidum*, the hybrid, *D. nobile* × *candidum* against FaDu cells, Table S1: Cell viabilities of the methanol extracts of the stems of 436 mutant lines of *D. nobile* × *candidum* at a concentration of 50 μg/mL in FaDu cells.
