# Supplementary material for: Chemical and Biological Profiles of Dendrobium in Two Different Species, Their Hybrid, and Gamma-Irradiated Mutant Lines of the Hybrid Based on LC-QToF MS and Cytotoxicity Analysis
Source: Plants (Basel). 2021 Jul 5;10(7):1376. doi: 10.3390/plants10071376 (PMC8309310; doi:10.3390/plants10071376)
Supplement: Supplementary file 1 [file plants-10-01376-s001.zip › plants-1273625-supplementary.pdf]

Supporting Information

Chemical profiles of Dendrobium in two different species and their hybrid and gamma-irradiated mutant lines of the hybrid by UPLC-QTOF MS

Bomi Nam [1,5†], Hyun-Jae Jang [2,†], Ah-Reum Han [1,†], Ye-Ram Kim [1], Chang Hyun Jin [1], Chan-Hun Jung [3], Kyo Bin Kang [4], Sang Hoon Kim [1], Min Jeong Hong [1], Jin-Baek Kim [1] and Hyung Won Ryu [2,*]

[1] Advanced Radiation Technology Institute, Korea Atomic Energy Research Institute (KAERI), Jeongeup-si, Jeollabuk-do 56212, Republic of Korea

[2] Natural Medicine Research Center, Korea Research Institute of Bioscience & Biotechnology (KRIBB), Cheongju-si, Chungbuk-do 28116, Republic of Korea

[3] Jeonju AgroBio-Materials Institute, Jeonju-si, Jeollabuk-do 54810, Republic of Korea

[4] Research Institute of Pharmaceutical Sciences, College of Pharmacy, Sookmyung Women's University, Seoul 04310, Korea

[5] Current address: Institute of Natural Cosmetic Industry for Namwon, Namwon-si, Jeollabuk-do 55801, Republic of Korea

* Correspondence: ryuhw@kribb.re.kr (H.W.R.); Tel. +82-43-240-6117 (H.W.R.)

† These authors contributed equally to this work.

CONTENS

Figure S1. ESI QTof MS spectrum of gigantol (peak 1)

Figure S2. ESI QTof MS spectrum of (1*R*,2*R*)-1,7-hydroxy-2,8-methoxy-2,3-dihydrophenanthrene-4(1*H*)-one (peak 2)

Figure S3. ESI QTof MS spectrum of tristin (peak 3)

Figure S4. ESI QTof MS spectrum of (-)-syringaresinol (peak 4)

Figure S5. ESI QTof MS spectrum of lusianthridin (peak 5)

Figure S6. ESI QTof MS spectrum of 2,7-dihydroxy-phenanthrene-1,4-dione (peak 6)

Figure S7. ESI QTof MS spectrum of densiflorol B (peak 7)

Figure S8. ESI QTof MS spectrum of denthyrsinin (peak 8)

Figure S9. ESI QTof MS spectrum of moscatilin (peak 9)

Figure S10. ESI QTof MS spectrum of lusianthridin dimer (peak 10)

Figure S11. ESI QTof MS spectrum of batatasin III (peak 11)

Figure S12. ESI QTof MS spectrum of ephemeranthol A (peak 12)

Figure S13. ESI QTof MS spectrum of thunalbene (peak 13)

Figure S14. ESI QTof MS spectrum of dehydroorchinol (peak 14)

Figure S15. ESI QTof MS spectrum of dendrobine (peak 15)

Figure S16. ESI QTof MS spectrum of shihunine (peak 16)

Figure S17. ESI QTof MS spectrum of 1,5,7-trimethoxy-2-phenanthrenol (peak 17)

Figure S18. ESI QTof MS spectrum of differential metabolite (M1)

Figure S19. ESI QTof MS spectrum of differential metabolite (M2)

Figure S20. Cytotoxicities of the methanol extracts of Dendrobii Herba, *D. nobile*, *D. candidum*, the hybrid, *D. nobile* × *candidum* against FaDu cells.

Table S1. Cell viabilities of the methanol extracts of the stems of 436 mutant lines of *D. nobile* × *candidum* at a concentration of 50 μg/ml in FaDu cells

Figure S1. ESI QTof MS spectrum of gigantol (peak 1)

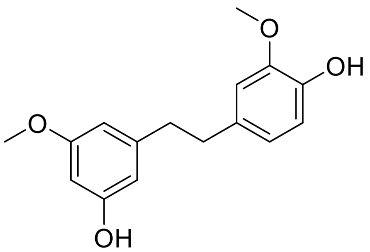

Chemical Formula: $C_{16}H_{18}O_4$
Molecular Weight: 274.3160

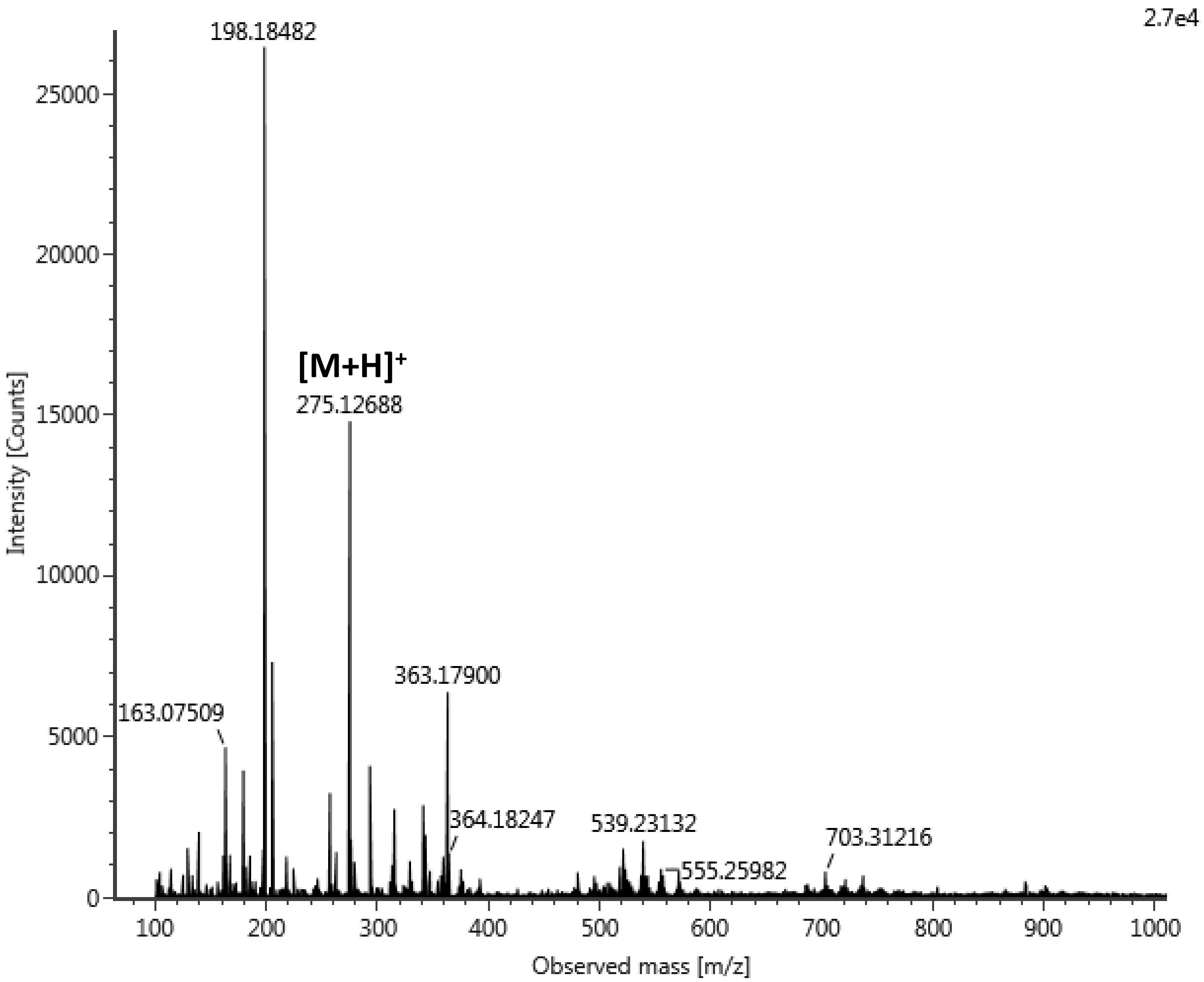

Composition	i-FIT Confidence (%)	m/z RMS (PPM)	Intensity RMS (%)	Predicted m/z	m/z error (PPM)	m/z error (mDa)	DBE
C16H18O4	100.000000	2.062404	10.146742	275.127786	-2.135983	-0.585515	8.000000

Figure S2. **ESI QTof MS spectrum of (1R,2R)-1,7-hydroxy-2,8-methoxy-2,3-dihydrophenanthrene-4(1H)-one (peak 2)**

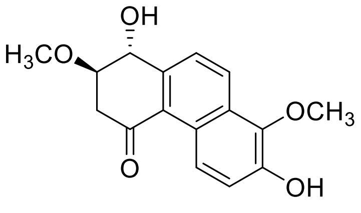

Chemical Formula: $C_{16}H_{16}O_5$
Molecular Weight: 288.2990

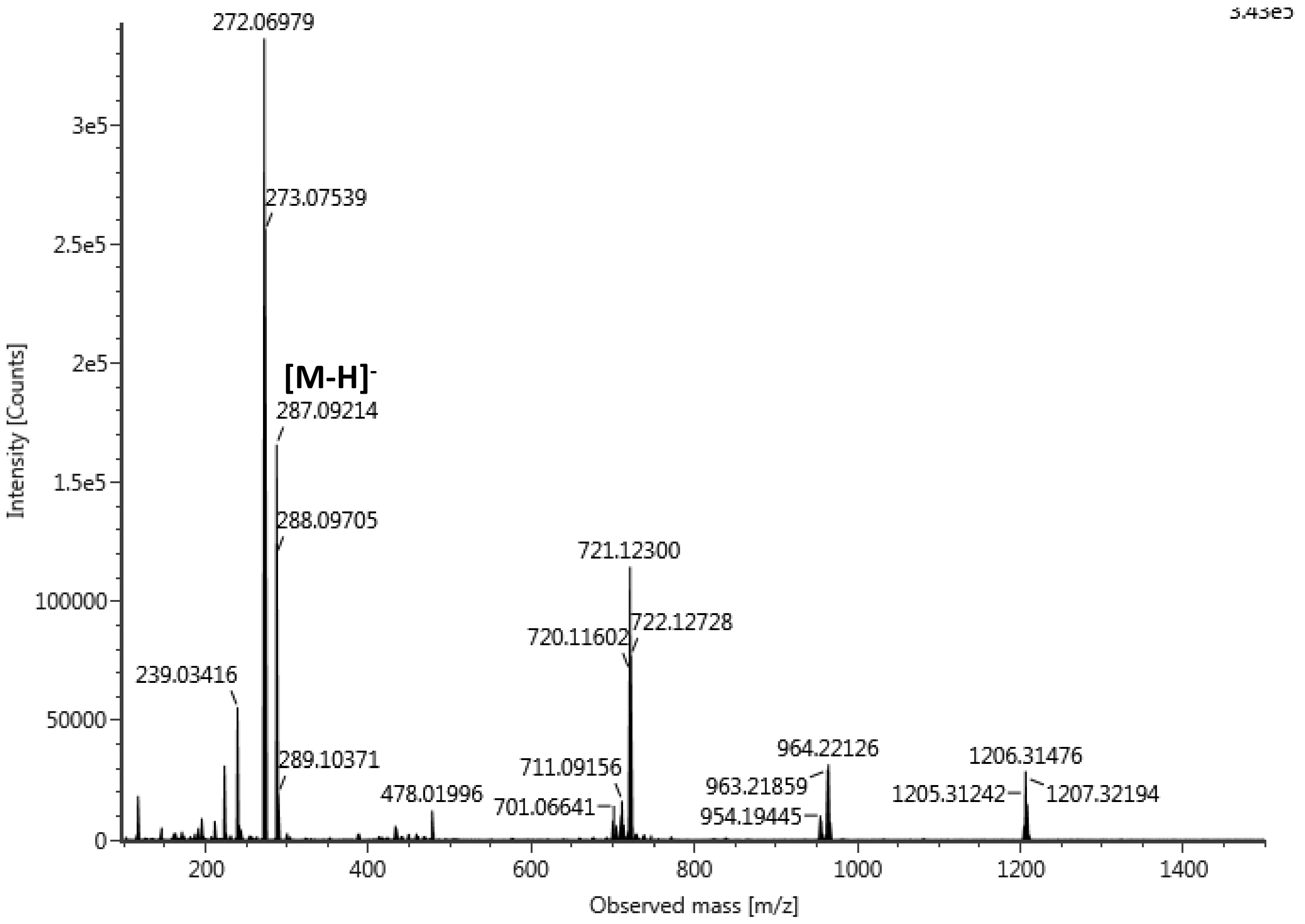

Composition	i-FIT Confidence (%)	m/z RMS (PPM)	Intensity RMS (%)	Predicted m/z	m/z error (PPM)	m/z error (mDa)	DBE
C16H14O5	100.000000	6.845093	134.530461	287.091400	4.439199	1.269990	10.000000

Figure S3. ESI QTof MS spectrum of tristin (peak 3)

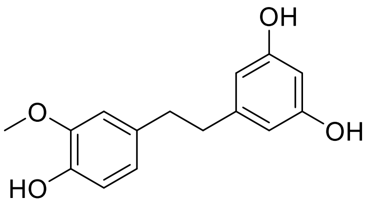

Chemical Formula: $C_{15}H_{16}O_4$
Molecular Weight: 260.2890

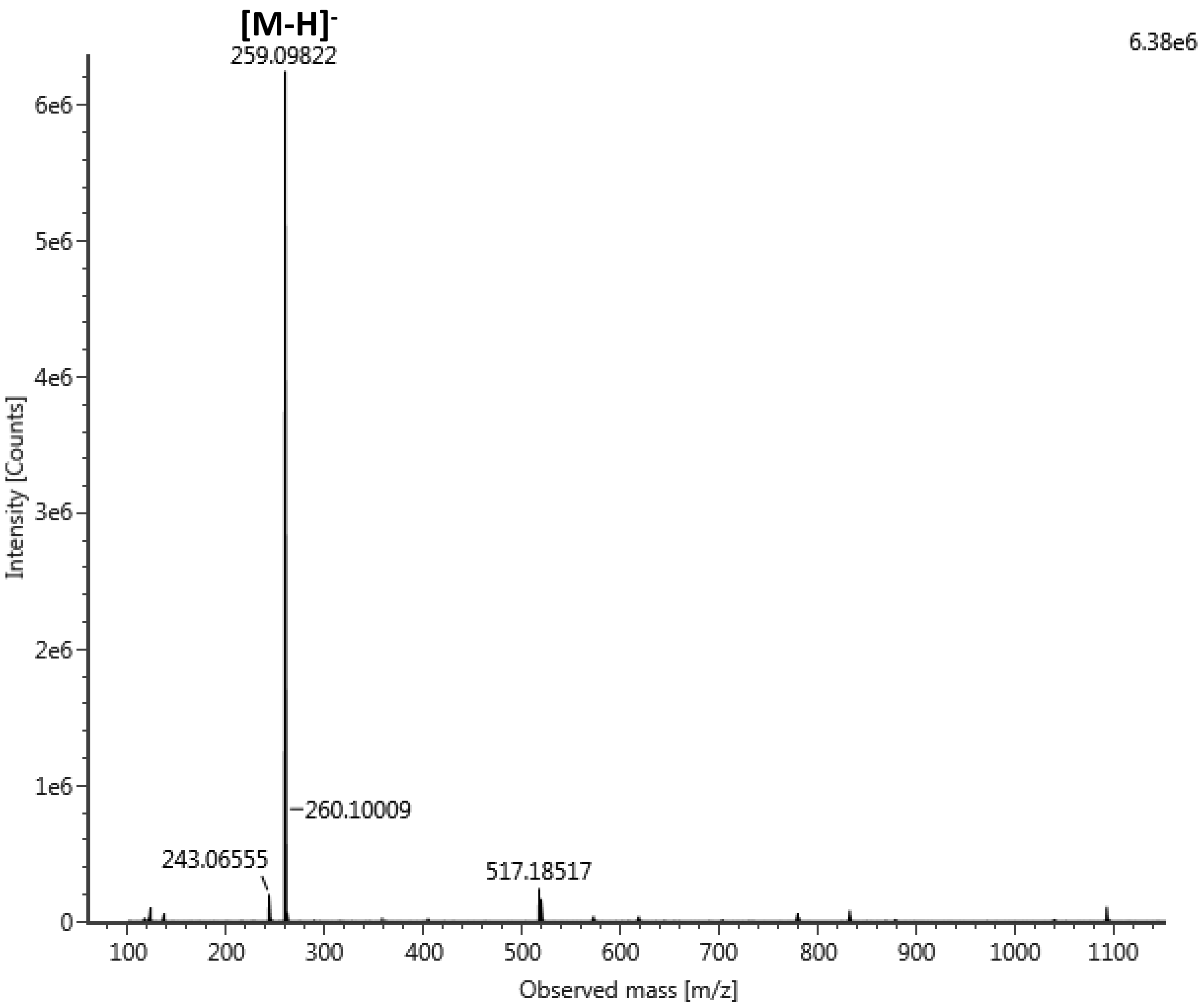

Composition	i-FIT Confidence (%)	m/z RMS (PPM)	Intensity RMS (%)	Predicted m/z	m/z error (PPM)	m/z error (mDa)	DBE
C15H14O4	99.999999	5.250568	0.548064	259.096485	5.171101	1.334612	9.000000

Figure S4. **ESI QTof MS spectrum of (-)-syringaresinol (peak 4)**

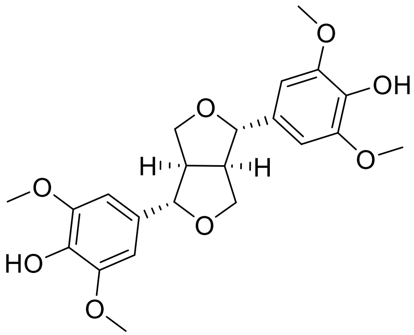

Chemical Formula: $C_{22}H_{26}O_8$
Molecular Weight: 418.4420

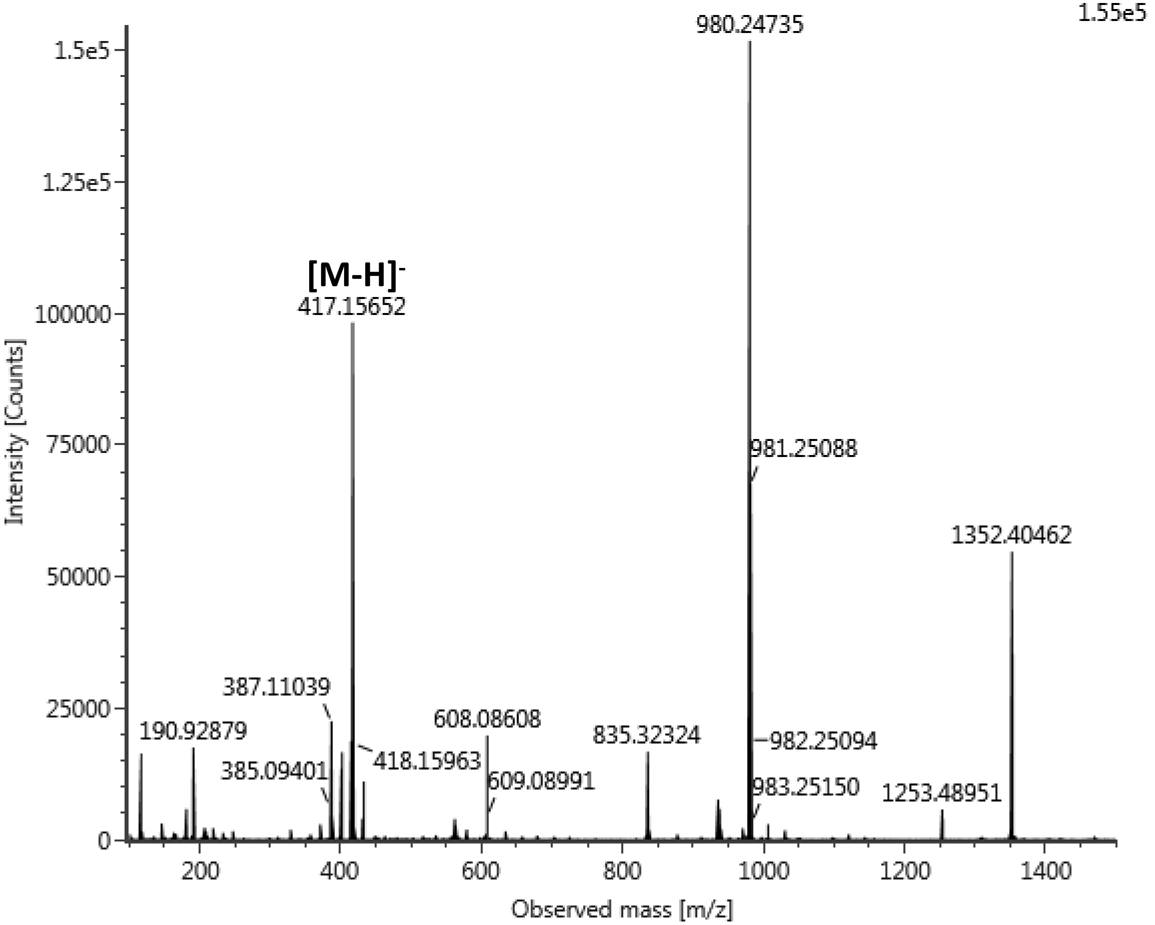

Composition	i-FIT Confidence (%)	m/z RMS (PPM)	Intensity RMS (%)	Predicted m/z	m/z error (PPM)	m/z error (mDa)	DBE
C22H26O8	99.995099	5.182257	1.556060	417.155491	5.210012	2.178644	10.000000

Figure S5. **ESI QTof MS spectrum of lusianthridin (peak 5)**

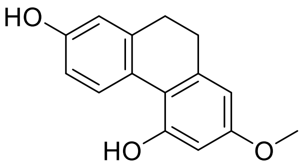

Chemical Formula: $C_{15}H_{14}O_3$
Molecular Weight: 242.2740

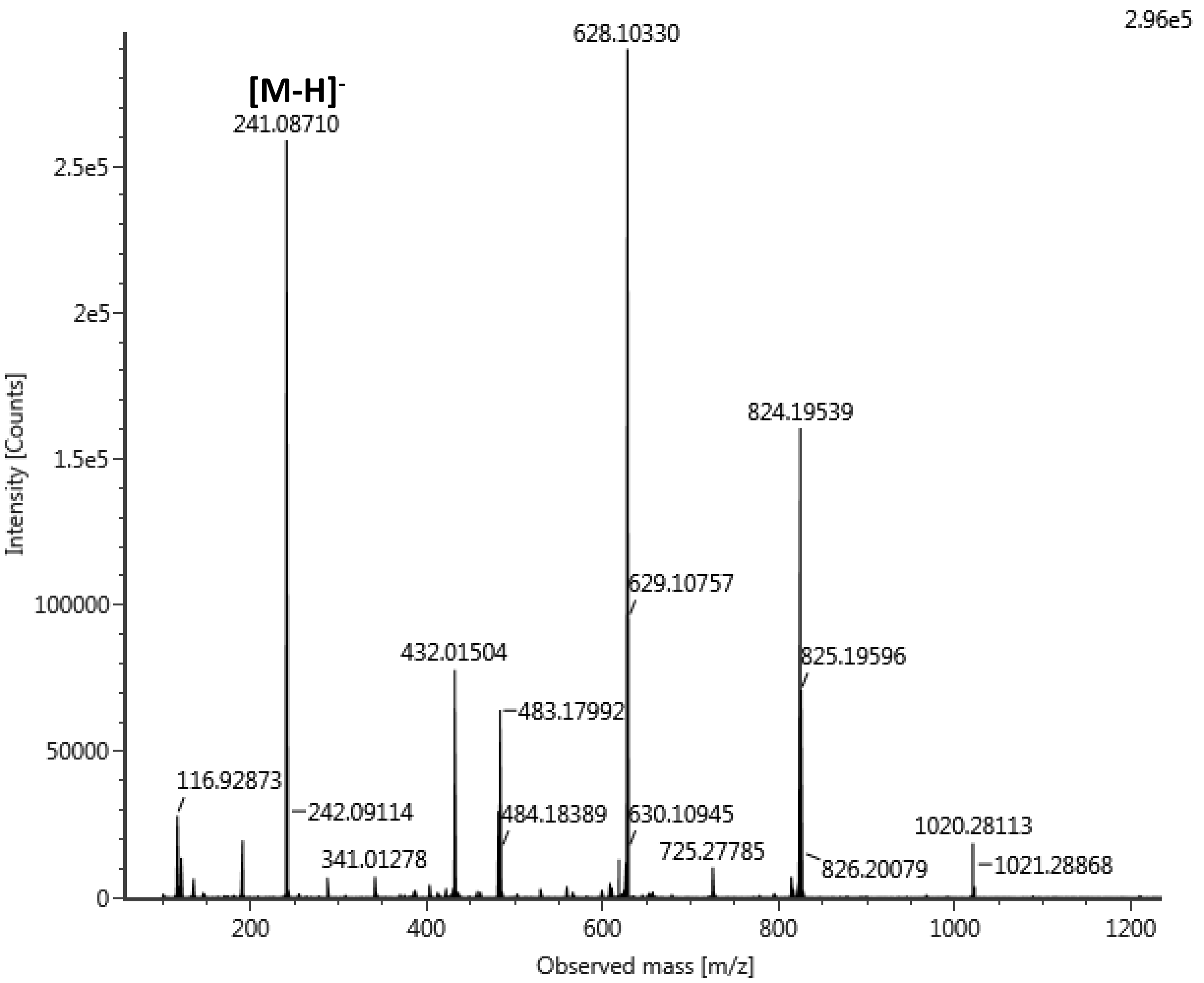

Composition	i-FIT Confidence (%)	m/z RMS (PPM)	Intensity RMS (%)	Predicted m/z	m/z error (PPM)	m/z error (mDa)	DBE
C15H14O3	99.999999	2.352884	1.287014	241.087018	0.834957	0.202138	9.000000

Figure S6. **ESI QTof MS spectrum of 2,7-dihydroxy-phenanthrene-1,4-dione (peak 6)**

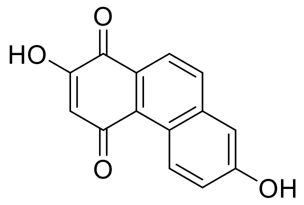

Chemical Formula: $C_{14}H_8O_4$
Molecular Weight: 240.2140

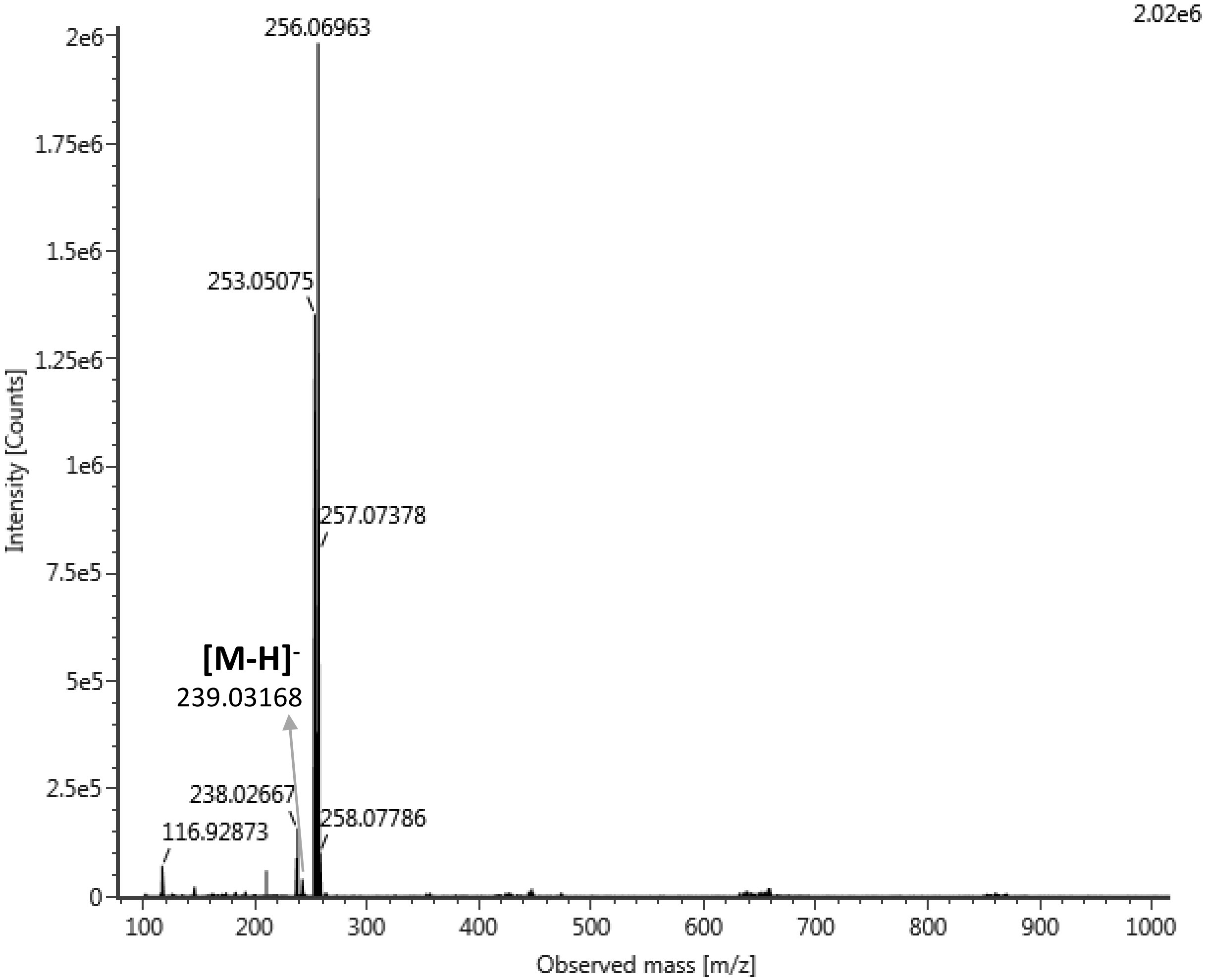

Composition	i-FIT Confidence (%)	m/z RMS (PPM)	Intensity RMS (%)	Predicted m/z	m/z error (PPM)	m/z error (mDa)	DBE
C14H6O4	100.000000	8.698541	3.834344	239.033885	-9.264313	-2.205133	12.000000

Figure S7. ESI QTof MS spectrum of densiflorol B (peak 7)

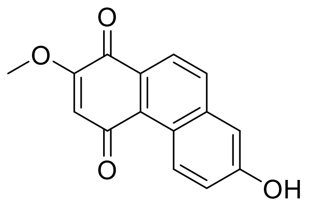

Chemical Formula: $C_{15}H_{10}O_4$
Molecular Weight: 254.2410

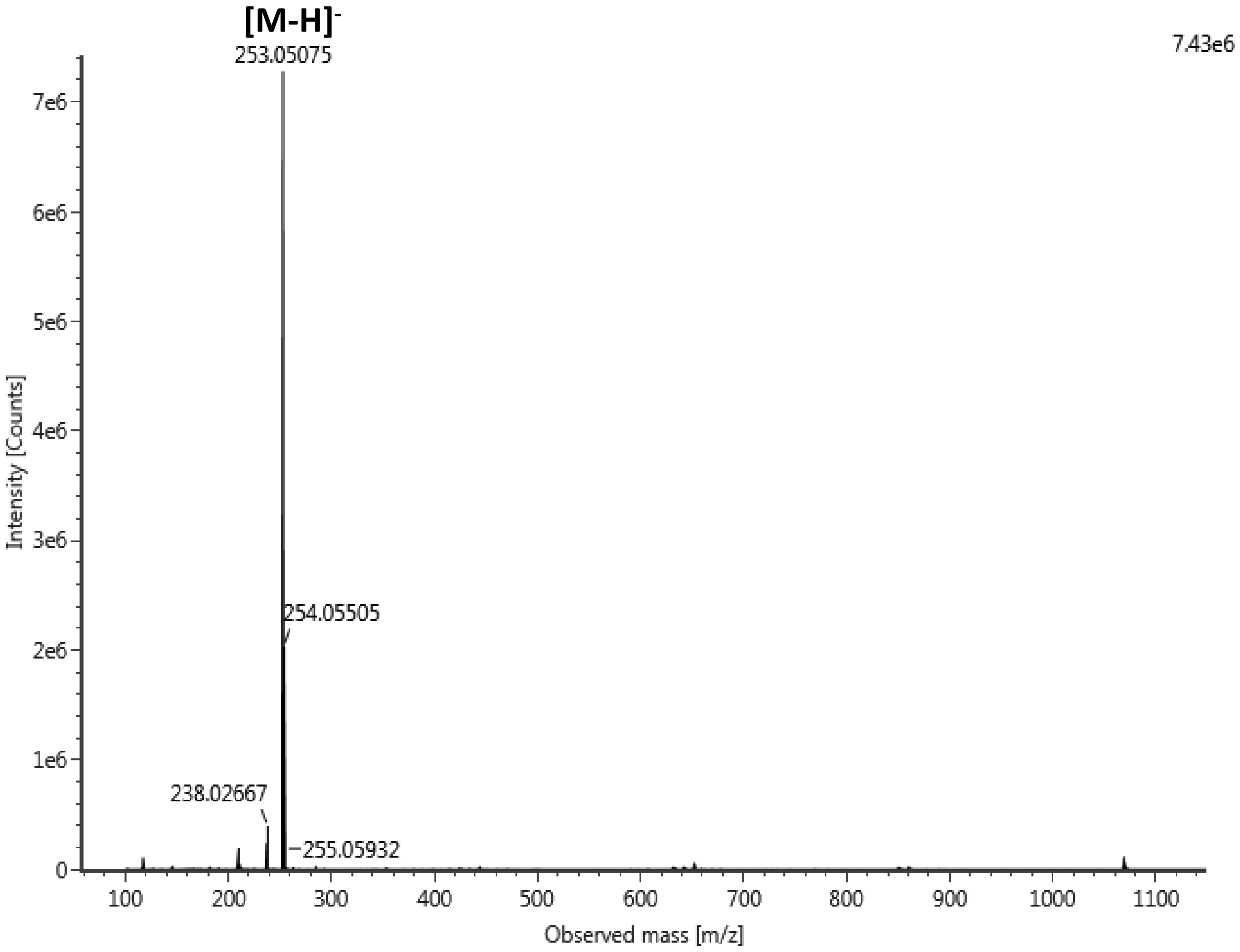

Composition	i-FIT Confidence (%)	m/z RMS (PPM)	Intensity RMS (%)	Predicted m/z	m/z error (PPM)	m/z error (mDa)	DBE
C15H8O4	100.000000	6.354830	34.999835	253.049535	4.105658	1.034804	12.000000

Figure S8. **ESI QTof MS spectrum of denthyrsinin (peak 8)**

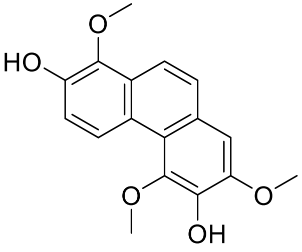

Chemical Formula: $C_{17}H_{16}O_5$
Molecular Weight: 300.3100

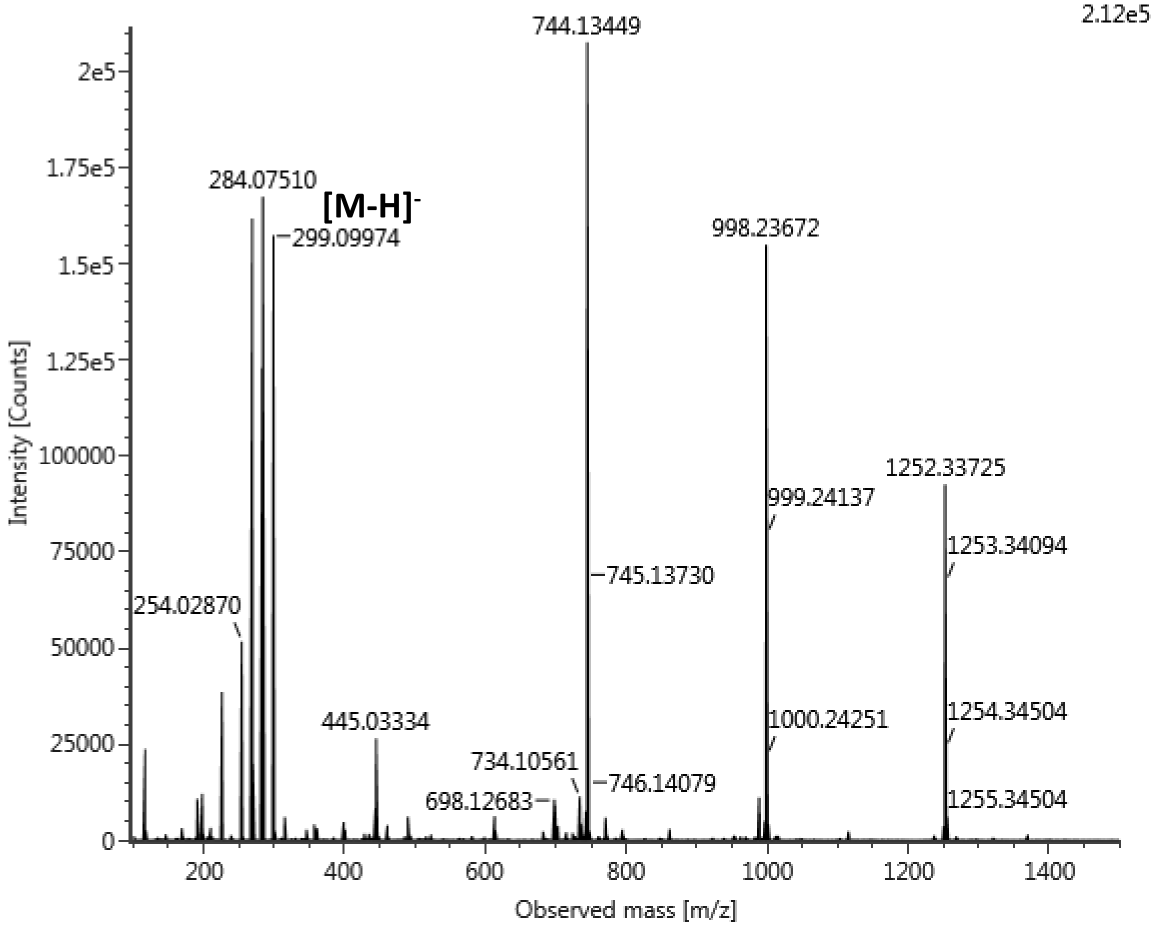

Composition	i-FIT Confidence (%)	m/z RMS (PPM)	Intensity RMS (%)	Predicted m/z	m/z error (PPM)	m/z error (mDa)	DBE
C17H16O5	99.701890	30.880711	1.946832	299.092497	22.734689	6.822830	10.000000
C10H20O10	0.298110	12.401611	17.005385	299.098370	3.164179	0.949591	1.000000

Figure S9. ESI QTof MS spectrum of moscatilin (peak 9)

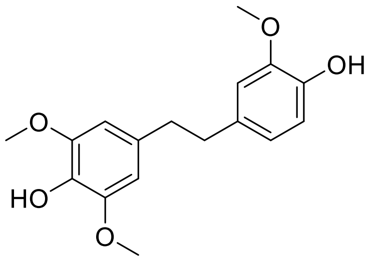

Chemical Formula: $C_{17}H_{20}O_5$
Molecular Weight: 304.3420

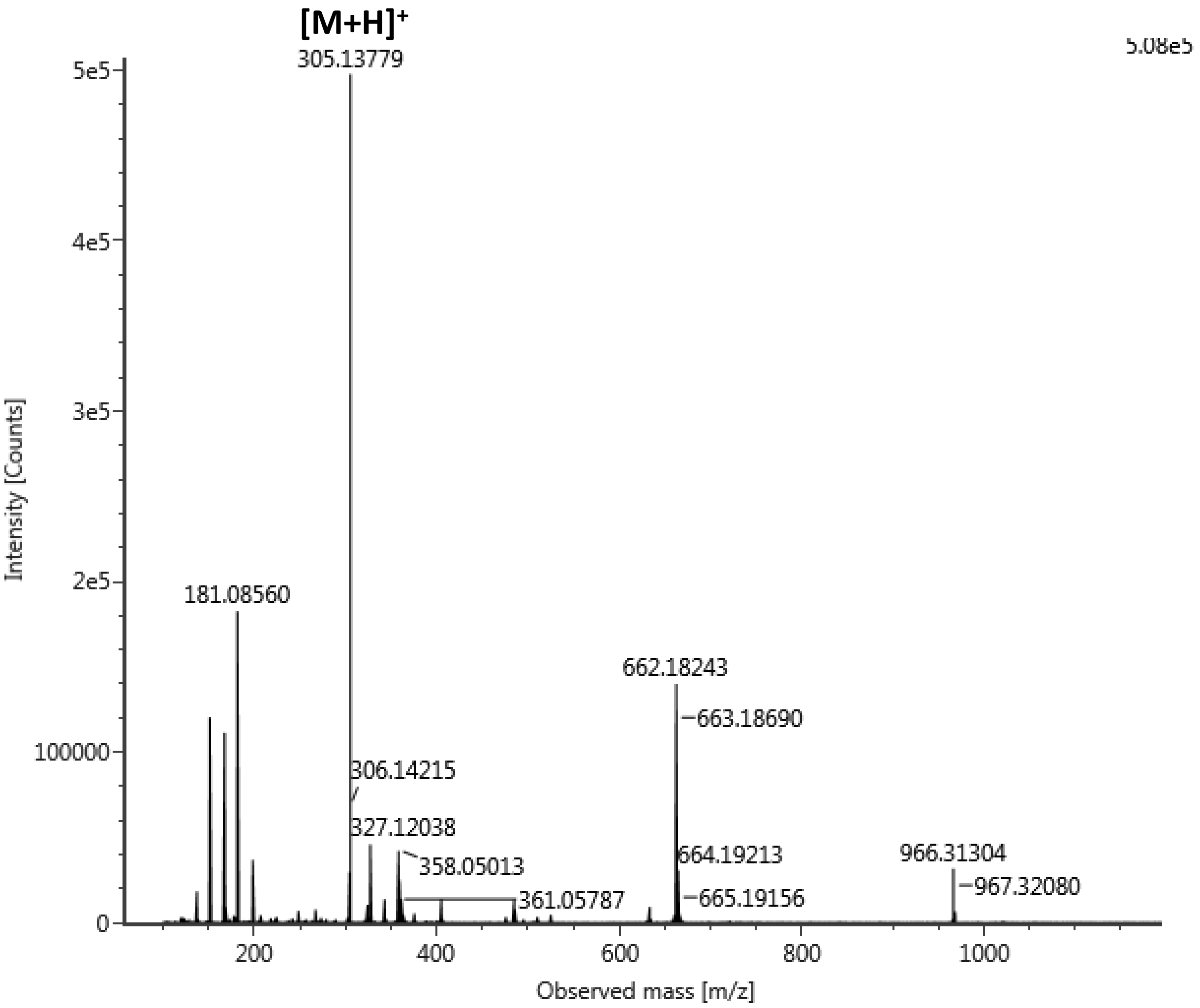

Composition	i-FIT Confidence (%)	m/z RMS (PPM)	Intensity RMS (%)	Predicted m/z	m/z error (PPM)	m/z error (mDa)	DBE
C17H20O5	99.999597	4.143328	2.003800	305.138350	-3.518895	-1.070201	8.000000

Figure S10. ESI QTof MS spectrum of lusianthridin dimer (peak 10)

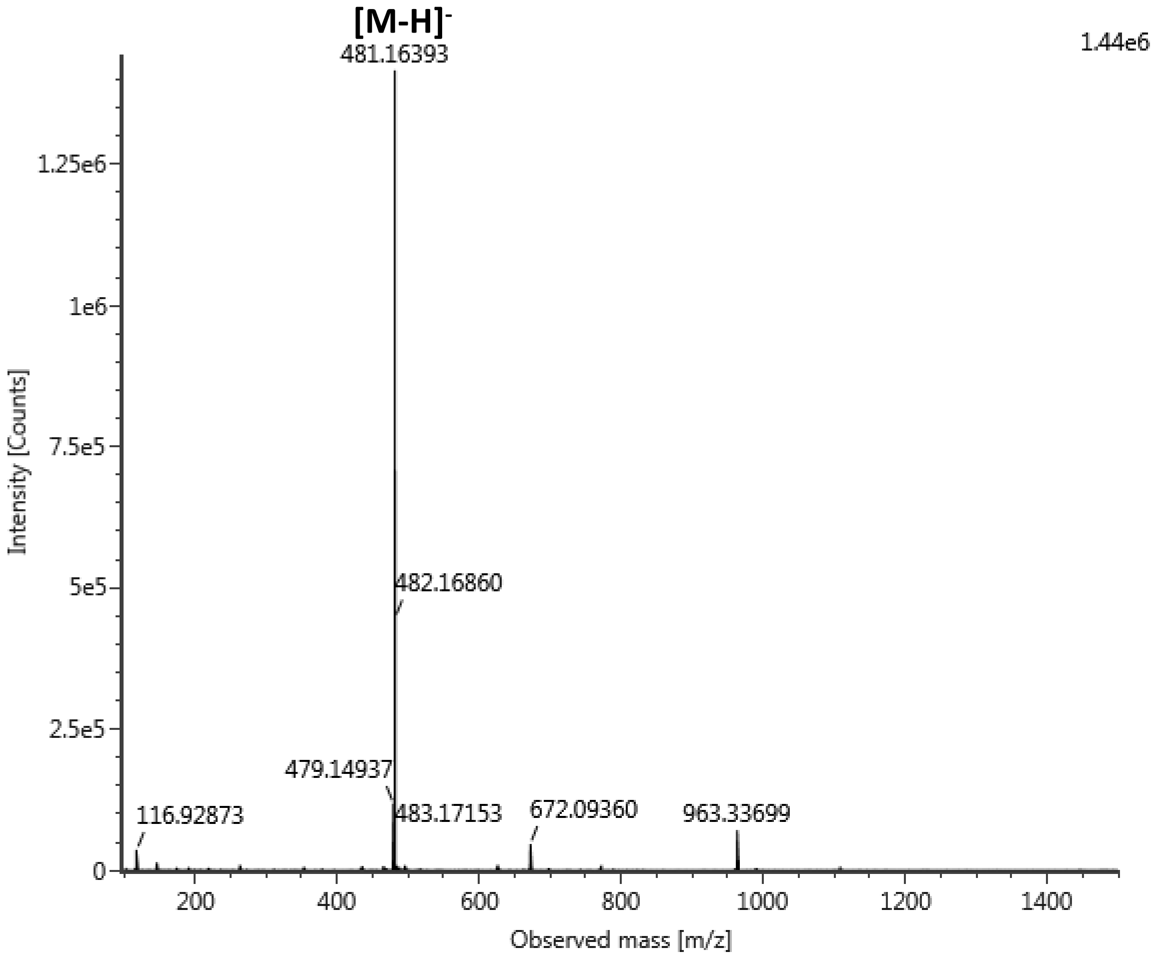

Composition	i-FIT Confidence (%)	m/z RMS (PPM)	Intensity RMS (%)	Predicted m/z	m/z error (PPM)	m/z error (mDa)	DBE
C30H26O6	100.000000	4.065246	8.823027	481.165662	-4.670776	-2.252111	18.000000

Figure S11. ESI QTof MS spectrum of batatasin III (peak 11)

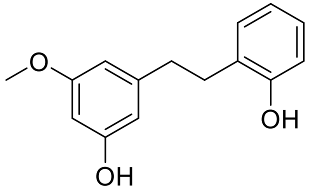

Chemical Formula: $C_{15}H_{16}O_3$
Molecular Weight: 244.2900

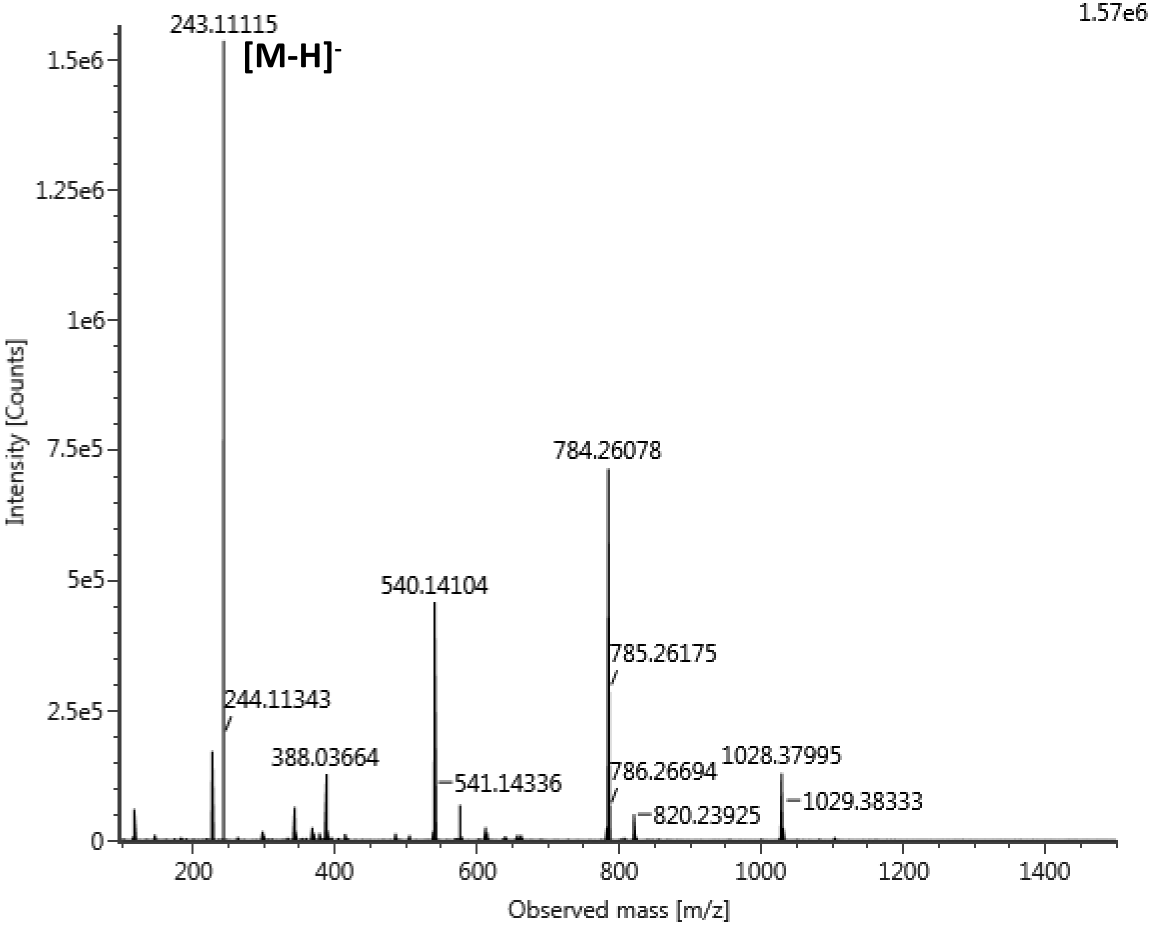

Composition	i-FIT Confidence (%)	m/z RMS (PPM)	Intensity RMS (%)	Predicted m/z	m/z error (PPM)	m/z error (mDa)	DBE
C15H16O3	99.994514	30.697427	0.877315	243.102668	31.182004	7.612075	8.000000

Figure S12. ESI QTof MS spectrum of ephemeranthol A (peak 12)

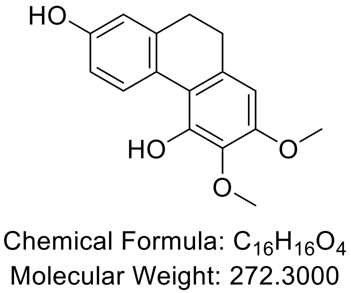

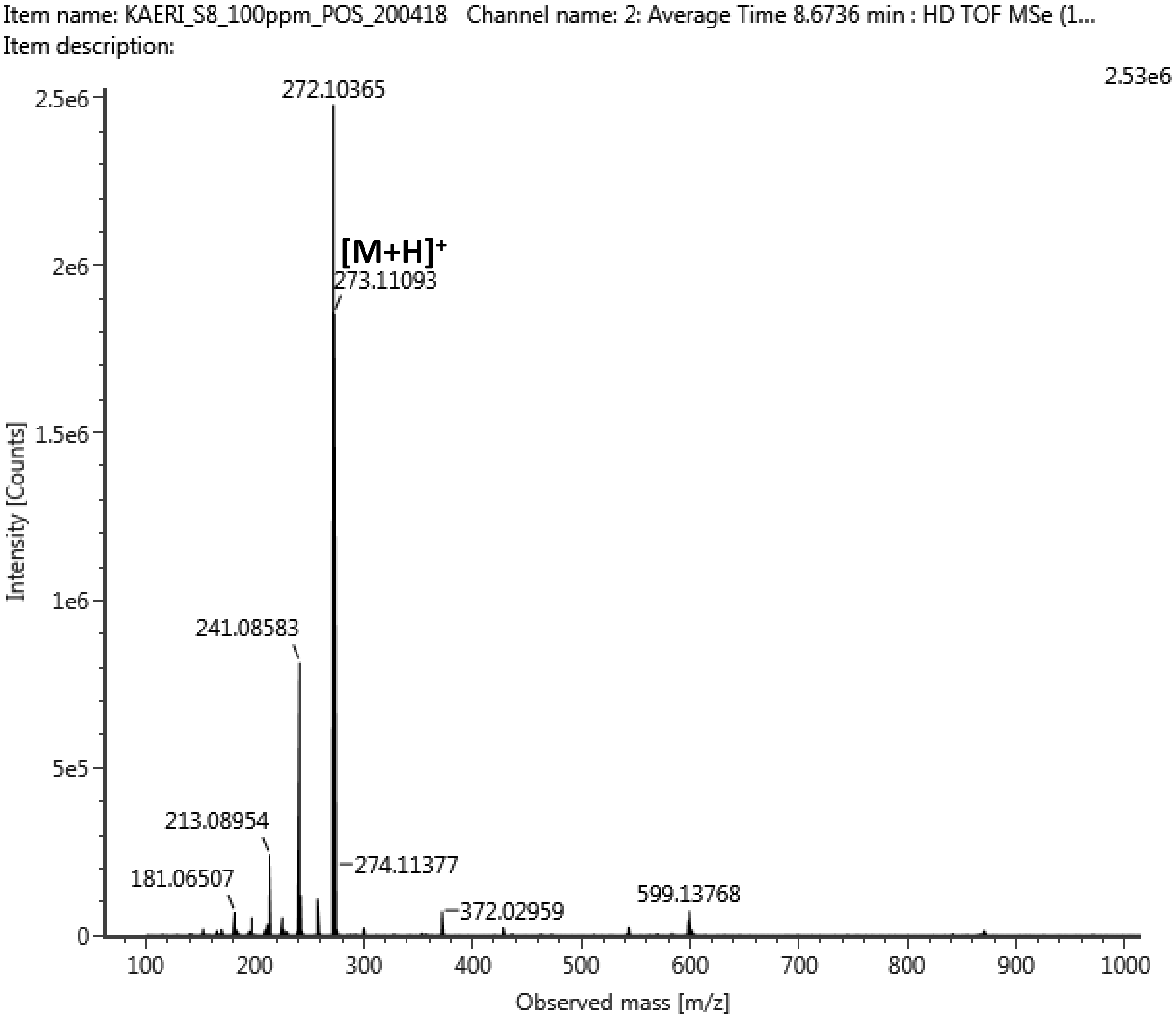

Composition	i-FIT Confidence (%)	m/z RMS (PPM)	Intensity RMS (%)	Predicted m/z	m/z error (PPM)	m/z error (mDa)	DBE
C16H16O4	99.997773	3.910662	1.906610	273.112135	3.912257	1.064548	9.000000

Figure S13. **ESI QTof MS spectrum of thunalbene (peak 13)**

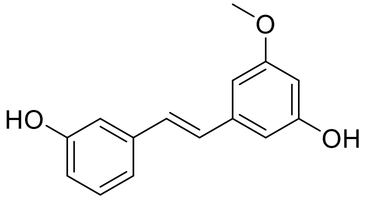

Chemical Formula: $C_{15}H_{14}O_3$
Molecular Weight: 242.2740

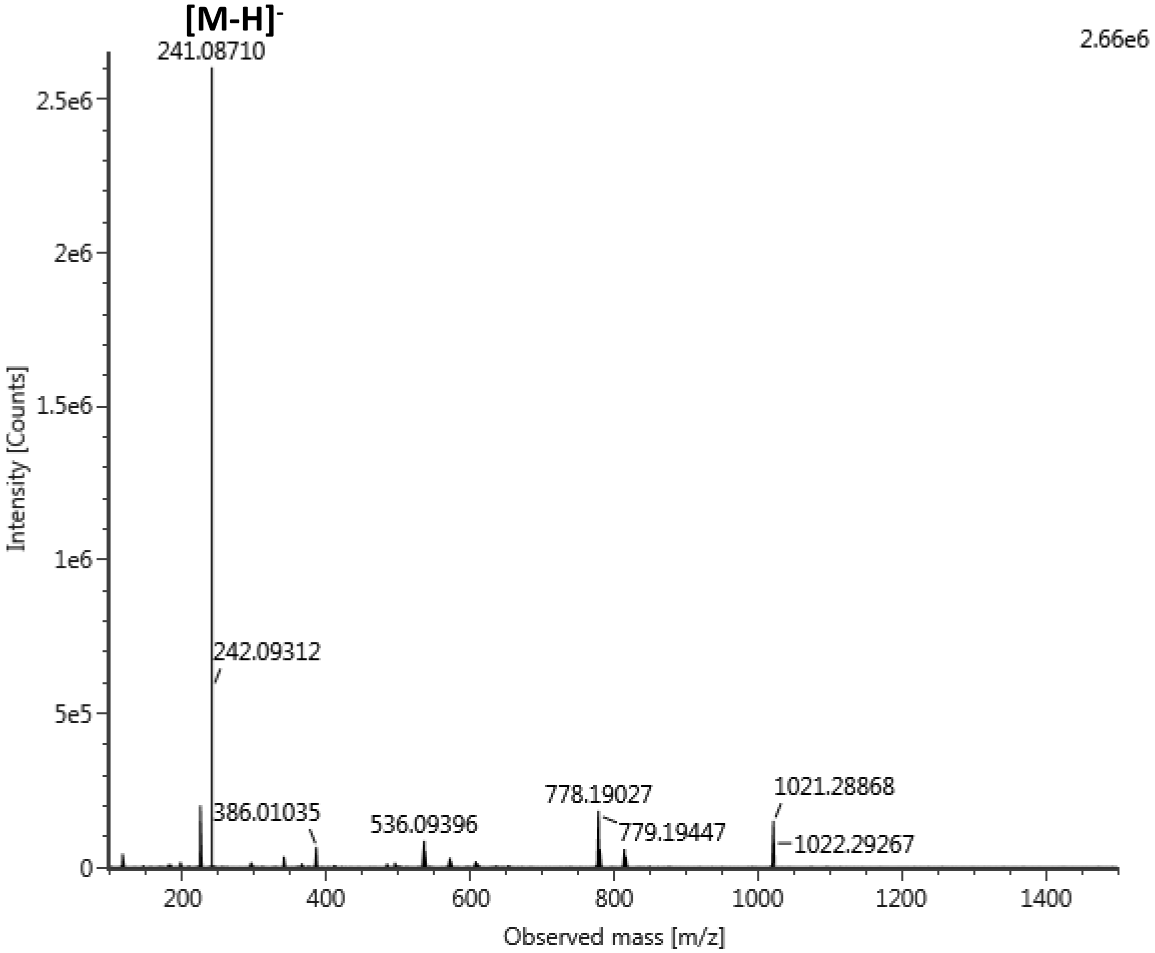

Composition	i-FIT Confidence (%)	m/z RMS (PPM)	Intensity RMS (%)	Predicted m/z	m/z error (PPM)	m/z error (mDa)	DBE
C15H14O3	100.000000	3.587588	29.224711	241.087018	1.330631	0.322138	9.000000

Figure S14. ESI QTof MS spectrum of dehydroorchinol (peak 14)

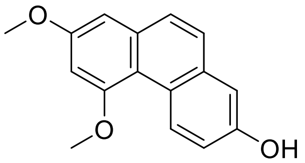

Chemical Formula: $C_{16}H_{14}O_3$
Molecular Weight: 254.2850

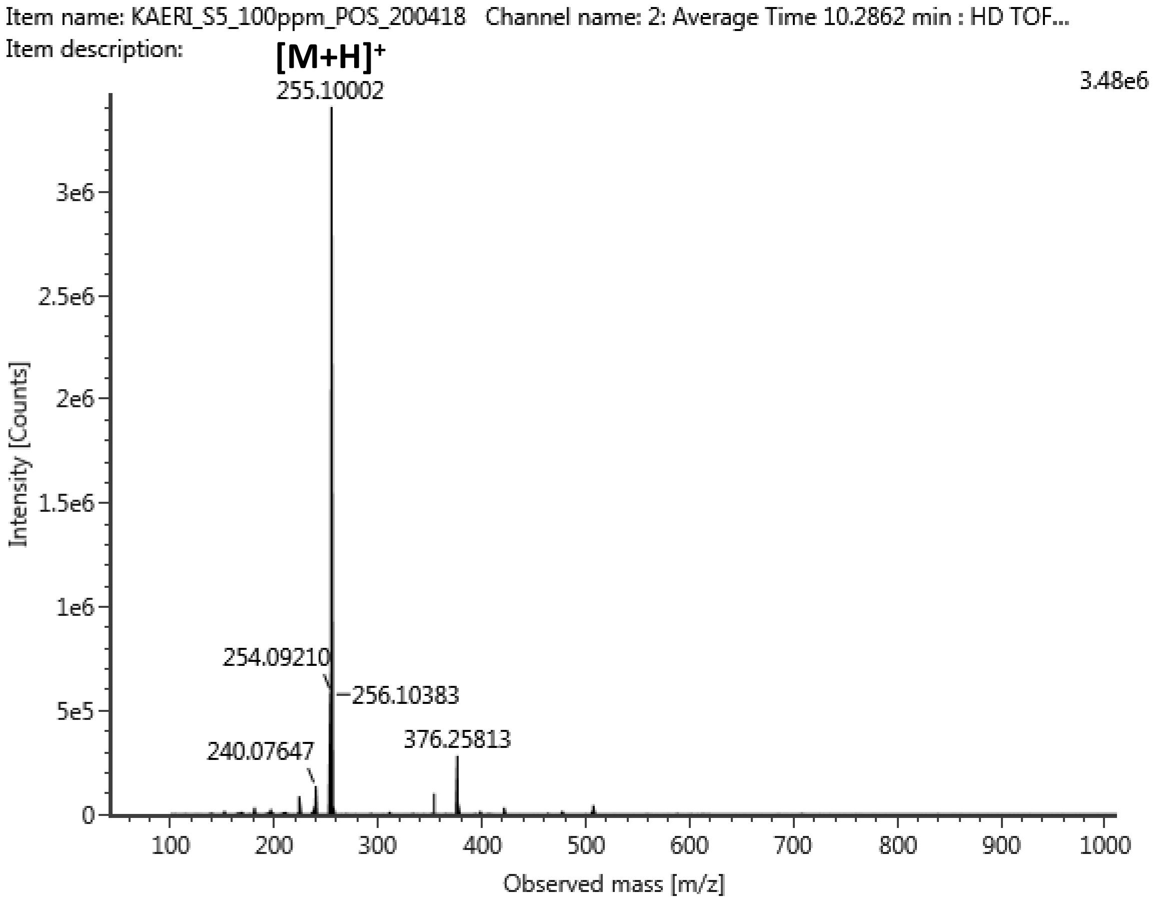

Composition	i-FIT Confidence (%)	m/z RMS (PPM)	Intensity RMS (%)	Predicted m/z	m/z error (PPM)	m/z error (mDa)	DBE
C16H14O3	100.000000	2.349546	1.715586	255.101571	-2.206924	-0.560765	10.000000

Figure S15. **ESI QTof MS spectrum of dendrobine (peak 15)**

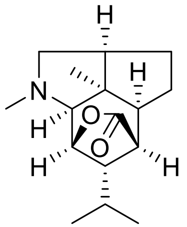

Chemical Formula: $C_{16}H_{25}NO_2$
Molecular Weight: 263.3810

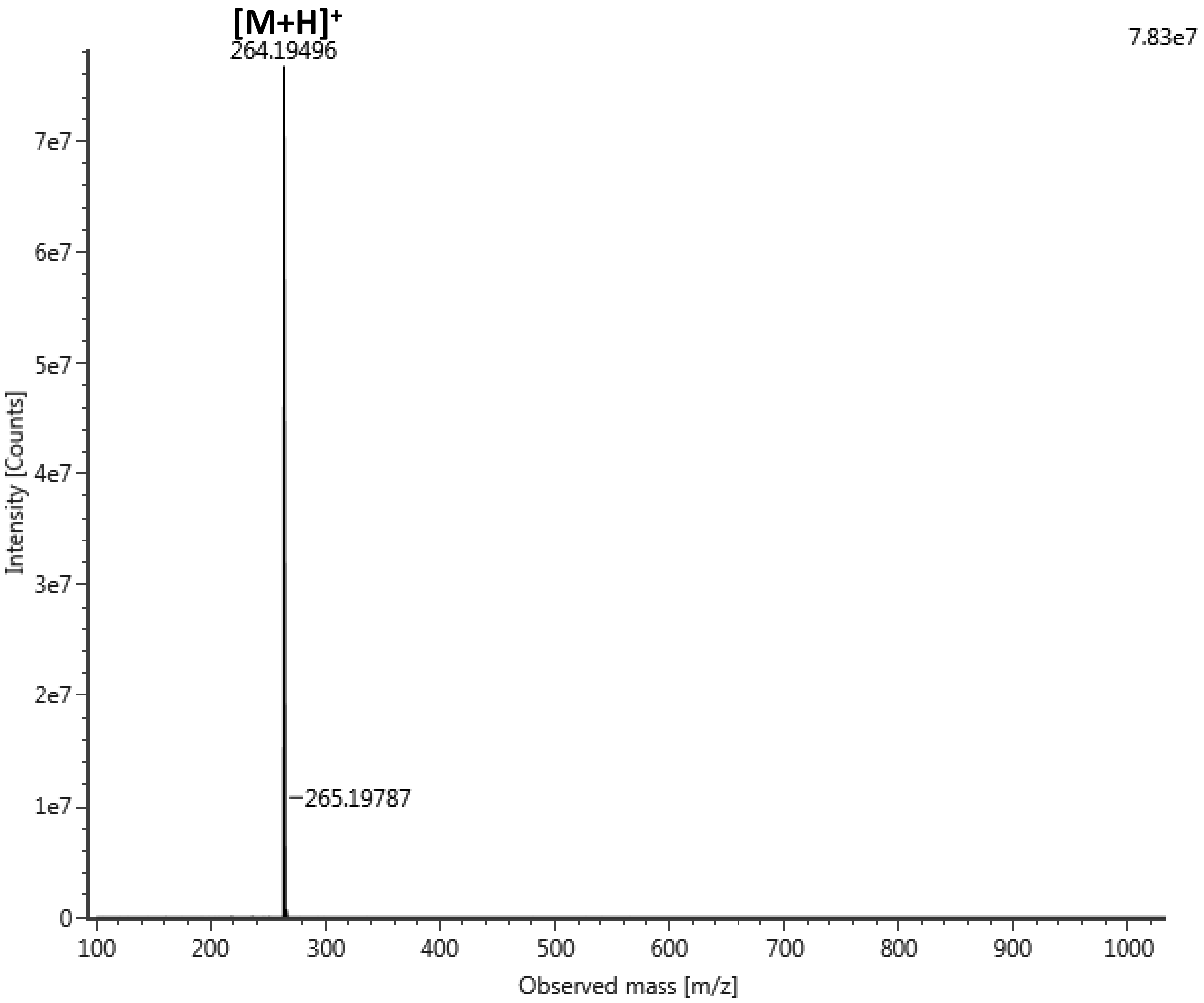

Composition	i-FIT Confidence (%)	m/z RMS (PPM)	Intensity RMS (%)	Predicted m/z	m/z error (PPM)	m/z error (mDa)	DBE
C16H25NO2	100.000000	1.874275	2.901497	264.195806	-2.034673	-0.535501	5.000000

Figure S16. **ESI QTof MS spectrum of shihunine (peak 16)**

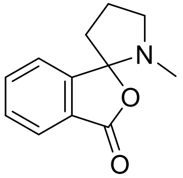

Chemical Formula: $C_{12}H_{13}NO_2$
Molecular Weight: 203.2410

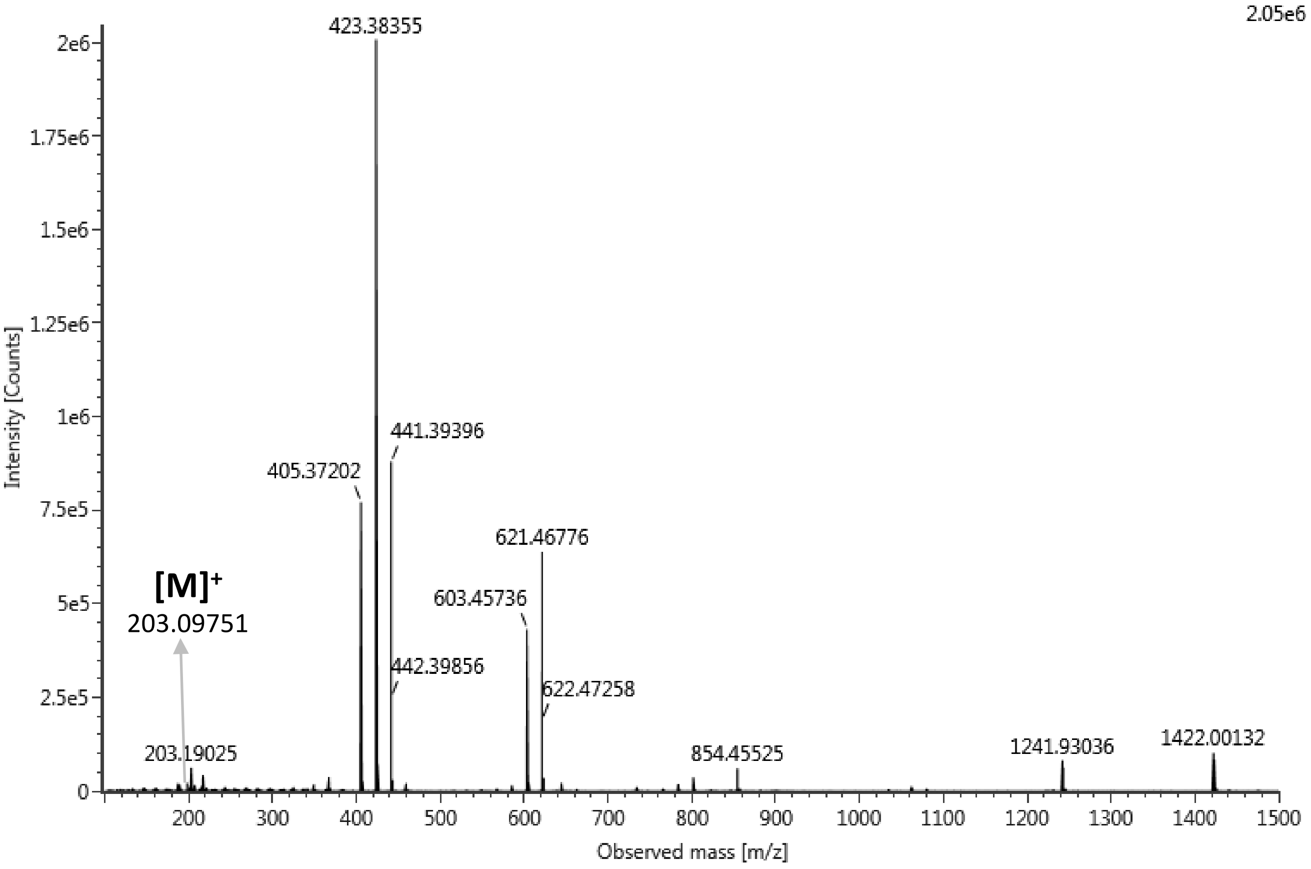

Composition	i-FIT Confidence (%)	m/z RMS (PPM)	Intensity RMS (%)	Predicted m/z	m/z error (PPM)	m/z error (mDa)	DBE
C12H13NO2	100.000000	415.075256	113.161722	203.095177	11.485910	2.332753	7.000000

Figure S17. **ESI QTof MS spectrum of 1,5,7-trimethoxy-2-phenanthrenol (peak 17)**

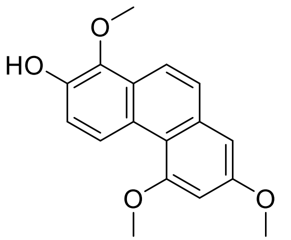

Chemical Formula: $C_{17}H_{16}O_4$
Molecular Weight: 284.3110

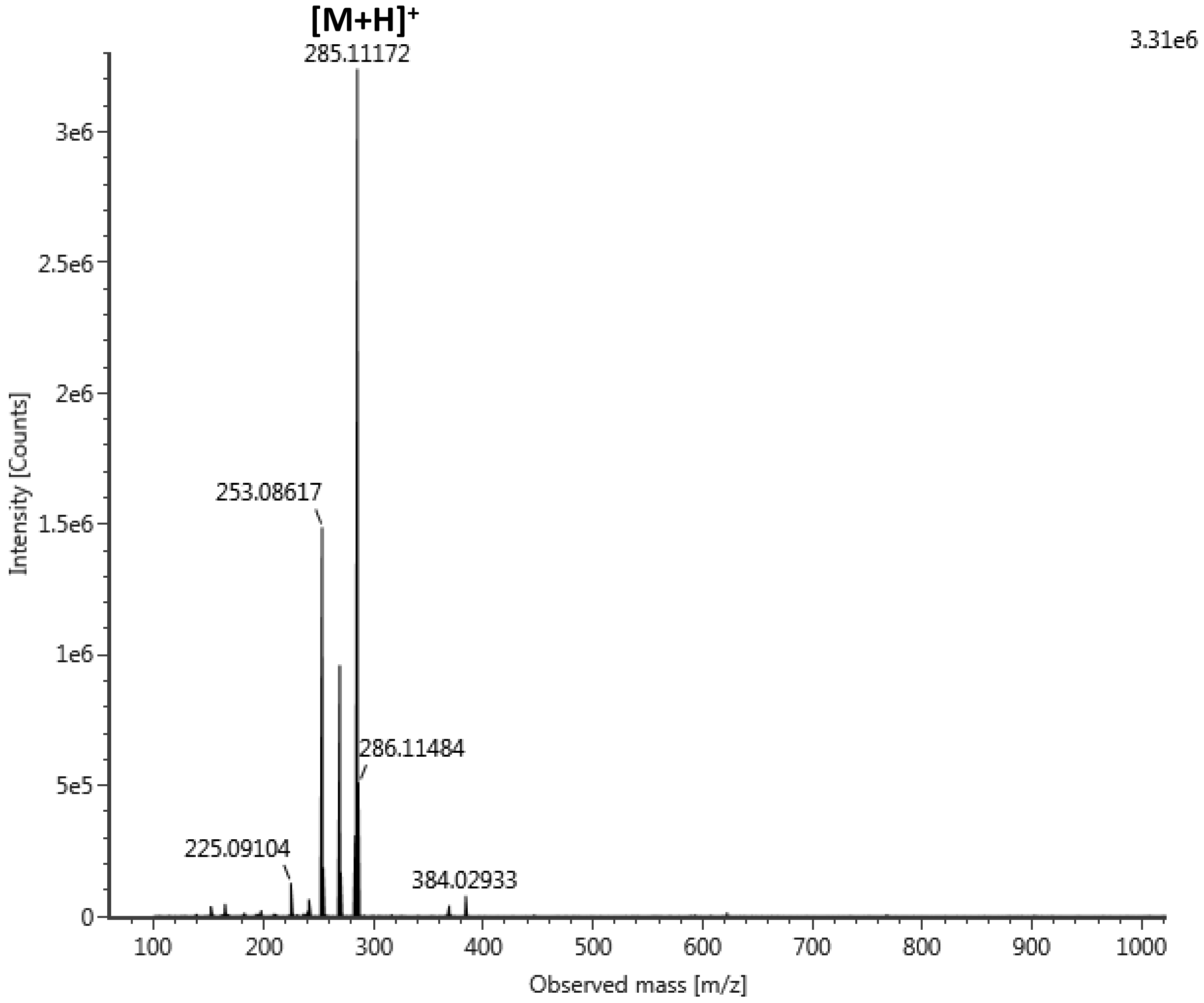

Composition	i-FIT Confidence (%)	m/z RMS (PPM)	Intensity RMS (%)	Predicted m/z	m/z error (PPM)	m/z error (mDa)	DBE
C17H16O4	100.000000	5.294233	0.890622	285.112135	-5.404553	-1.535452	10.000000

Figure S18. ESI QTof MS spectrum of differential metabolite (M1)

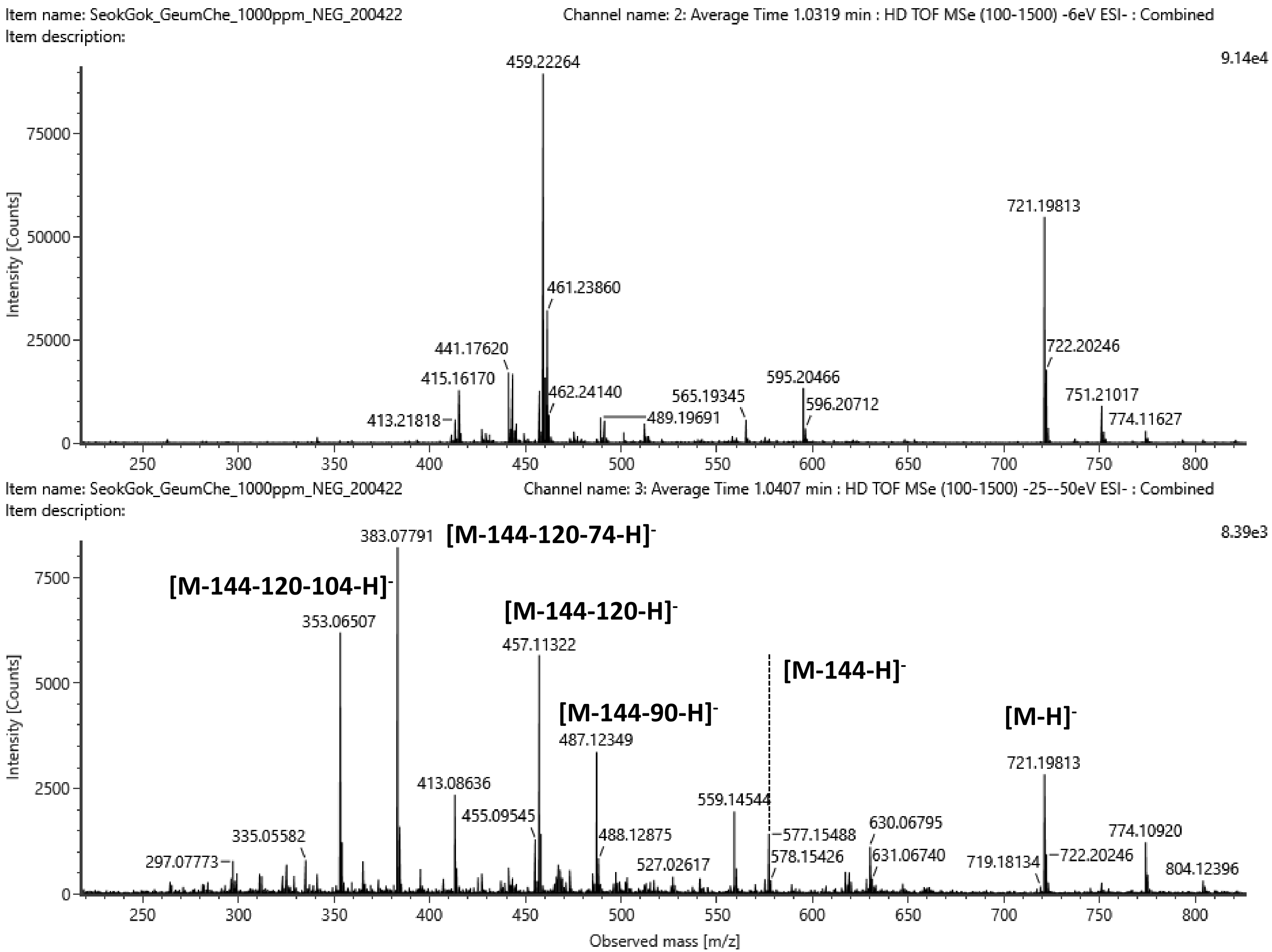

Composition	i-FIT Confidence (%)	m/z RMS (PPM)	Intensity RMS (%)	Predicted m/z	m/z error (PPM)	m/z error (mDa)
C33H38O18	100.000000	0.797720	4.134514	721.198538	-0.564883	-0.407962

Figure S19. ESI QTof MS spectrum of differential metabolite (M2)

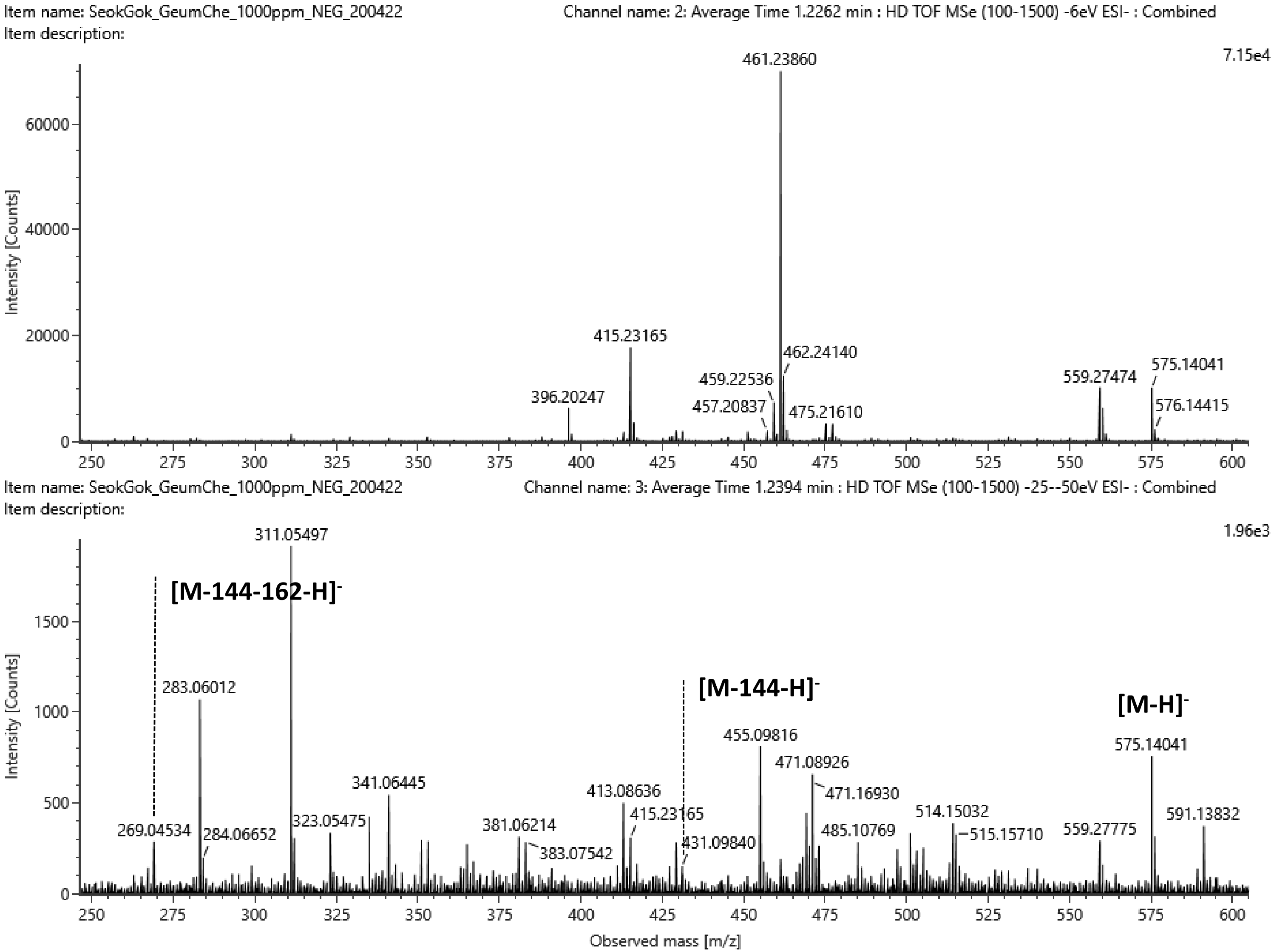

Composition	i-FIT Confidence (%)	m/z RMS (PPM)	Intensity RMS (%)	Predicted m/z	m/z error (PPM)	m/z error (mDa)
C27H28O14	47.352255	6.784584	6.786975	575.140629	-0.380377	-0.219153

Figure S20. Cytotoxicities of the methanol extracts of Dendrobii Herba, *D. nobile*, *D. candidum*, the hybrid, *D. nobile × candidum* against FaDu cells. Values are presented as the mean ± SD of three independent experiments.

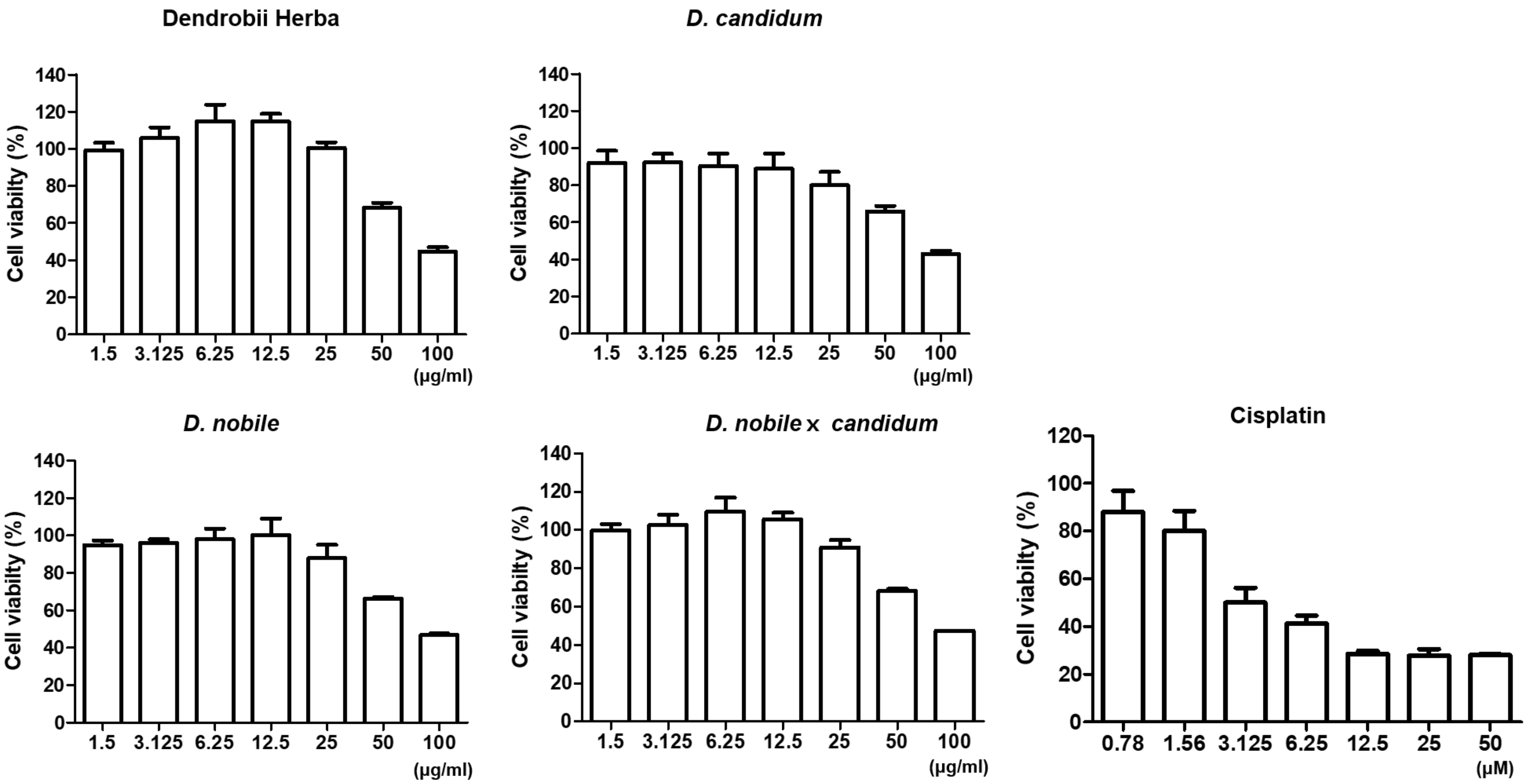

Table S1. Cell viabilities of the methanol extracts of the stems of 436 mutant lines of *D. nobile* × *candidum* at a concentration of 50 μg/ml in FaDu cells

No.	Viability(%)	SD	No.	Viability(%)	SD	No.	Viability(%)	SD	No.	Viability(%)	SD	No.	Viability(%)	SD	No.	Viability(%)	SD	No.	Viability(%)	SD	No.	Viability(%)	SD	No.	Viability(%)	SD
**2**	65.10	0.48	**68**	55.68	1.78	**159**	68.29	1.97	**220**	54.27	2.43	**275**	54.32	2.24	**343**	61.95	1.74	**424**	63.90	0.43	**525**	35.25	3.06	**587**	54.64	3.47
**7**	60.35	0.60	**77**	40.64	3.44	**162**	62.47	1.46	**222**	65.33	1.99	**282**	44.86	1.92	**345**	64.46	2.99	**434**	51.76	1.96	**528**	34.82	0.71	**591**	60.03	3.47
**11**	62.31	0.98	**80**	69.88	0.77	**164**	55.08	7.99	**223**	65.33	1.80	**285**	50.16	0.66	**348**	53.42	1.72	**440**	59.74	1.61	**531**	56.22	1.78	**593**	60.64	1.63
**13**	62.69	0.69	**86**	64.01	1.22	**166**	54.22	6.69	**224**	65.39	2.27	**286**	46.70	2.44	**358**	52.83	1.22	**441**	57.49	3.00	**532**	54.22	0.93	**595**	60.82	0.66
**14**	63.88	2.55	**89**	66.85	1.47	**169**	63.39	1.66	**227**	66.11	2.07	**287**	64.18	1.57	**359**	53.83	1.14	**443**	51.84	1.46	**533**	53.69	0.62	**597**	61.77	3.18
**15**	63.93	3.32	**91**	63.92	2.81	**172**	63.61	1.10	**231**	61.95	0.26	**289**	69.44	1.83	**360**	63.23	22.29	**453**	62.71	4.14	**536**	51.21	2.32	**599**	59.22	0.87
**18**	64.23	1.26	**92**	60.07	1.62	**173**	63.90	1.76	**233**	56.95	2.56	**290**	64.11	2.15	**362**	64.33	0.67	**460**	57.82	5.40	**544**	47.51	2.50	**600**	59.16	1.22
**24**	61.94	2.86	**93**	56.97	2.55	**174**	63.33	2.46	**239**	53.47	2.84	**292**	60.17	2.57	**363**	61.06	1.19	**473**	53.94	3.61	**545**	55.63	0.67	**601**	52.78	3.76
**29**	61.74	3.22	**95**	66.85	2.04	**176**	61.62	0.99	**243**	62.03	2.56	**295**	55.91	0.79	**364**	55.01	3.77	**478**	59.33	5.06	**547**	56.15	4.13	**612**	56.47	1.12
**30**	66.04	2.91	**98**	66.78	3.65	**181**	58.50	2.59	**244**	55.80	2.28	**296**	48.36	1.92	**366**	56.17	2.69	**485**	72.76	2.58	**549**	54.50	1.81	**616**	51.90	2.03
**31**	65.39	2.87	**103**	65.58	3.30	**182**	59.54	3.13	**246**	53.29	3.62	**304**	58.25	0.97	**372**	64.56	6.10	**488**	64.36	2.49	**550**	51.32	3.79	**619**	50.80	1.05
**39**	68.43	1.02	**104**	64.29	4.31	**183**	53.44	1.76	**252**	61.35	1.50	**310**	62.32	1.92	**374**	62.93	2.44	**490**	58.45	2.18	**552**	52.91	3.35	**620**	51.52	1.10
**42**	69.89	4.56	**111**	62.33	3.15	**185**	66.89	11.85	**254**	63.21	2.55	**311**	56.90	2.78	**379**	55.85	0.19	**496**	61.47	0.92	**557**	53.53	2.84	**623**	45.72	1.64
**43**	70.03	4.31	**114**	56.73	2.51	**187**	63.55	1.04	**256**	60.86	3.81	**321**	73.15	4.43	**386**	51.75	2.13	**498**	67.88	2.29	**558**	59.17	1.93	**630**	58.28	2.88
**46**	70.16	4.75	**115**	59.45	1.20	**191**	52.10	2.98	**257**	60.21	1.11	**325**	63.01	1.31	**387**	52.35	2.91	**502**	57.88	6.63	**560**	54.52	1.25	**632**	63.87	0.97
**48**	69.35	2.76	**120**	61.58	1.03	**195**	55.71	1.00	**260**	51.50	2.61	**326**	64.41	4.36	**393**	53.61	2.29	**506**	54.22	7.60	**562**	57.19	4.48	**639**	58.13	1.73
**54**	63.87	1.67	**124**	61.74	3.23	**198**	62.99	2.50	**262**	61.64	0.50	**327**	59.61	0.43	**394**	50.94	3.14	**508**	57.42	4.14	**563**	53.95	3.73	**642**	58.92	1.40
**55**	52.82	1.98	**131**	62.22	3.43	**199**	63.08	3.83	**263**	56.64	1.67	**330**	63.44	1.27	**398**	54.87	0.98	**514**	64.81	4.35	**564**	47.54	4.76	**643**	63.63	1.25
**60**	57.45	2.22	**140**	66.24	1.65	**201**	62.70	3.78	**264**	60.88	3.05	**331**	55.40	0.60	**400**	55.92	1.66	**515**	65.35	3.24	**566**	44.73	1.83	**651**	55.69	5.82
**63**	60.15	3.65	**141**	66.94	2.26	**204**	60.80	1.46	**269**	54.28	2.44	**336**	69.36	2.39	**404**	53.65	2.28	**516**	64.63	0.88	**568**	47.68	2.17	**654**	63.92	3.70
**64**	59.50	3.22	**143**	66.75	2.55	**210**	61.61	6.98	**270**	48.88	4.36	**337**	68.43	2.24	**407**	61.80	5.66	**517**	59.42	2.52	**570**	57.19	0.29	**655**	62.56	2.12
**65**	59.18	2.42	**145**	65.69	2.63	**211**	61.13	1.92	**272**	61.40	1.27	**338**	69.70	4.03	**408**	60.23	0.71	**519**	38.18	1.85	**572**	57.57	2.16	**657**	56.39	0.39
**66**	58.00	2.35	**149**	64.59	0.95	**215**	58.50	2.01	**273**	52.69	0.87	**339**	66.85	0.74	**409**	59.57	2.34	**521**	34.95	0.75	**574**	53.61	1.78	**658**	54.92	1.08
**67**	56.77	1.61	**157**	64.57	1.33	**217**	58.35	1.35	**274**	53.23	1.28	**341**	58.07	3.05	**423**	59.45	6.41	**524**	34.94	1.12	**575**	51.21	0.90	**661**	59.10	2.62

No.	Viability(%)	SD	No.	Viability(%)	SD	No.	Viability(%)	SD	No.	Viability(%)	SD	No.	Viability(%)	SD	No.	Viability(%)	SD	No.	Viability(%)	SD	No.	Viability(%)	SD	No.	Viability(%)	SD
**666**	51.74	0.76	**755**	57.10	0.13	**846**	55.55	0.89	**926**	57.37	2.56	**1007**	49.13	0.74	**1064**	51.82	1.47	**1138**	49.88	0.92	**1194**	59.26	4.20	**1275**	50.68	2.06
**667**	50.40	1.85	**757**	57.61	1.61	**849**	53.49	1.16	**929**	48.94	2.07	**1009**	48.45	1.91	**1066**	48.23	2.87	**1140**	50.41	4.78	**1196**	56.33	2.53	**1284**	51.19	2.09
**671**	54.39	0.81	**762**	55.68	2.03	**851**	55.36	2.00	**933**	53.16	4.29	**1011**	68.92	24.15	**1069**	41.44	3.98	**1143**	53.95	4.40	**1200**	64.35	2.53	**1285**	51.47	3.13
**673**	51.80	2.34	**765**	55.44	4.50	**857**	56.33	3.76	**936**	48.55	2.00	**1012**	53.05	1.88	**1072**	53.81	2.60	**1145**	52.98	3.62	**1205**	73.49	1.69	**1287**	59.27	0.99
**674**	54.89	1.62	**767**	61.41	4.45	**859**	55.68	0.90	**939**	48.75	3.32	**1015**	57.22	1.07	**1074**	51.82	1.33	**1147**	51.51	3.03	**1206**	76.36	3.02	**1302**	48.64	1.93
**676**	56.62	1.90	**777**	47.51	1.48	**863**	48.84	0.65	**946**	49.06	2.56	**1016**	56.58	3.47	**1080**	51.28	3.64	**1148**	52.77	5.34	**1209**	77.72	4.32	**1309**	44.44	1.70
**677**	58.80	2.79	**780**	56.34	1.94	**870**	56.76	2.76	**947**	51.01	1.37	**1017**	52.65	3.72	**1084**	46.74	5.09	**1153**	50.49	4.22	**1212**	73.32	5.84	**1312**	53.18	0.72
**681**	52.47	0.60	**793**	58.71	7.87	**871**	51.73	2.18	**954**	53.58	1.10	**1020**	51.49	3.06	**1089**	46.30	3.18	**1157**	46.63	0.40	**1215**	80.20	7.89	**1314**	51.93	2.40
**692**	54.46	2.19	**801**	48.93	2.10	**872**	51.96	2.25	**957**	49.23	0.63	**1021**	51.47	1.01	**1093**	41.49	1.34	**1162**	54.91	3.62	**1216**	72.73	1.67	**1317**	49.45	2.87
**696**	55.10	2.38	**802**	41.74	2.57	**875**	49.88	0.17	**960**	54.60	2.84	**1022**	47.79	2.22	**1096**	56.52	2.94	**1166**	57.42	3.88	**1218**	62.42	2.99	**1318**	53.23	3.48
**699**	60.24	11.57	**803**	57.07	1.07	**876**	42.58	1.94	**965**	59.36	3.58	**1026**	61.35	2.26	**1097**	54.05	3.72	**1167**	57.80	1.37	**1219**	60.53	2.69	**1320**	58.16	1.43
**706**	49.56	1.63	**804**	55.64	1.60	**878**	54.70	1.77	**966**	55.68	2.04	**1029**	56.60	2.08	**1098**	56.92	1.78	**1169**	57.97	5.94	**1220**	53.79	1.73	**1322**	51.47	0.85
**709**	53.90	1.26	**808**	46.42	5.70	**879**	50.25	2.24	**969**	49.48	0.58	**1032**	62.08	1.02	**1100**	53.17	1.60	**1172**	52.06	4.02	**1222**	57.79	0.35	**1326**	53.95	1.60
**710**	55.90	0.33	**810**	46.44	2.32	**885**	56.78	1.68	**971**	52.62	0.17	**1034**	53.70	1.24	**1101**	50.22	4.11	**1174**	61.52	1.93	**1223**	53.26	3.35	**1327**	50.37	1.50
**711**	60.74	1.81	**812**	42.56	3.02	**886**	53.48	2.70	**975**	57.47	1.12	**1041**	56.88	0.24	**1111**	59.38	0.58	**1175**	55.39	0.73	**1236**	54.50	6.28	**1329**	52.53	0.63
**715**	52.86	3.36	**814**	52.58	2.46	**895**	51.94	2.13	**978**	55.64	3.96	**1043**	53.66	2.68	**1115**	63.35	1.27	**1176**	56.16	2.08	**1238**	50.21	2.23	**1330**	48.64	1.93
**717**	60.27	0.48	**816**	55.35	3.75	**896**	65.86	3.54	**981**	50.24	3.92	**1044**	54.64	4.76	**1116**	64.67	0.62	**1178**	55.76	2.30	**1239**	77.45	0.81	**1331**	48.15	2.50
**721**	49.88	3.53	**818**	50.40	4.09	**902**	73.83	3.94	**986**	49.07	1.93	**1045**	56.62	4.50	**1117**	63.08	0.57	**1179**	57.34	2.75	**1252**	57.97	5.49	**1340**	53.18	0.72
**728**	51.04	0.93	**821**	55.76	1.05	**907**	55.48	3.94	**991**	53.69	1.65	**1052**	51.12	1.93	**1120**	64.77	0.72	**1180**	60.45	3.32	**1254**	44.79	4.20	**1341**	55.09	0.41
**729**	46.74	1.35	**822**	51.55	0.79	**908**	47.48	3.06	**993**	57.71	0.56	**1053**	47.56	3.39	**1121**	53.56	2.42	**1183**	59.18	2.71	**1256**	51.52	5.73	**1343**	51.93	2.40
**746**	55.64	4.78	**828**	53.51	2.24	**909**	53.45	2.93	**995**	60.55	1.13	**1059**	51.80	1.06	**1128**	60.70	3.06	**1187**	54.67	1.52	**1270**	81.52	9.55	**1346**	53.23	3.48
**747**	57.54	2.97	**831**	56.94	1.05	**918**	69.23	2.71	**998**	56.90	2.19	**1060**	55.93	1.57	**1130**	51.52	1.25	**1189**	51.75	2.88	**1271**	94.89	17.02	**1348**	58.16	1.43
**750**	64.01	1.42	**838**	61.29	4.39	**920**	65.92	4.50	**1003**	53.89	2.40	**1062**	56.75	2.99	**1131**	52.50	0.14	**1191**	75.33	3.10	**1273**	50.52	5.41	**1349**	52.84	1.36
**752**	59.46	1.24	**840**	55.44	1.18	**921**	63.53	3.01	**1005**	48.15	0.42	**1063**	56.88	1.61	**1134**	53.83	2.36	**1193**	60.56	2.61	**1274**	56.42	7.99	**1351**	49.22	1.61
																								**1352**	49.37	0.45
																								**1366**	53.95	1.60
																								**Untreated**	100.00	0.43
																								**DMSO**	93.95	2.42
																								**Cisplatine**	48.08	3.01
